# Serum peptidomic screening identified circulating peptide biomarkers predictive for preeclampsia

**DOI:** 10.3389/fcvm.2022.946433

**Published:** 2022-10-11

**Authors:** Shenglong Zhao, Chenghong Yin, Yanhong Zhai, Zhaoxia Jia, Shaofei Su, Yifan Lu, Lanlan Meng, Chunbo Li, Xiang Liu, Yuting Cong, Youran Li, Ying Liu, Lu Chen, Jing Wang, Zhengwen Xu, Yuanyuan Zheng, Zhi Sun, Ruben Y. Luo, Xiaobo Yu, He S. Yang, Xiaowei Liu, Zhen Zhao, Zheng Cao

**Affiliations:** ^1^Department of Obstetrics, Beijing Obstetrics and Gynecology Hospital, Beijing Maternal and Child Health Care Hospital, Capital Medical University, Beijing, China; ^2^Central Laboratory, Beijing Obstetrics and Gynecology Hospital, Beijing Maternal and Child Health Care Hospital, Capital Medical University, Beijing, China; ^3^Department of Laboratory Medicine, Beijing Obstetrics and Gynecology Hospital, Beijing Maternal and Child Health Care Hospital, Capital Medical University, Beijing, China; ^4^Center of Clinical Mass Spectrometry, Beijing Obstetrics and Gynecology Hospital, Beijing Maternal and Child Health Care Hospital, Capital Medical University, Beijing, China; ^5^Department of Information and Statistics, Beijing Obstetrics and Gynecology Hospital, Beijing Maternal and Child Health Care Hospital, Capital Medical University, Beijing, China; ^6^SCIEX, Shanghai, China; ^7^Department of Pharmacy, The First Affiliated Hospital of Zhengzhou University, Zhengzhou, China; ^8^Henan Engineering Research Center of Clinical Mass Spectrometry for Precision Medicine, Zhengzhou, China; ^9^Department of Pathology, School of Medicine, Stanford University, Stanford, CA, United States; ^10^State Key Laboratory of Proteomics, Beijing Proteome Research Center, National Center for Protein Sciences-Beijing (PHOENIX Center), Beijing Institute of Lifeomics, Beijing, China; ^11^Department of Pathology and Laboratory Medicine, Weill Cornell Medicine, New York, NY, United States

**Keywords:** preeclampsia, prediction, peptidomics, peptides, mass spectrometry

## Abstract

**Background:**

Reliable biomarkers are needed to improve preeclampsia (PE) prediction accuracy. With the investigational tool of peptidomics, we aimed to identify and validate potential serum peptide biomarkers in cohorts suspected for PE development in middle or late pregnancy.

**Methods:**

Totally 195 serum samples were prospectively collected from pregnant women with PE-related syndromes who were followed up for PE development until delivery. Serum peptidomic analysis was performed in the discovery cohort of 115 samples using matrix-assisted laser desorption ionization-time of flight coupled with Linear Trap Quadropole Orbitrap mass spectrometry. The candidate biomarkers were further validated using an in-house developed liquid chromatography tandem mass spectrometry (LC-MS/MS) method in an independent validation cohort of 80 serum samples.

**Results:**

We identified 8 peptides that were differentially expressed and originated from fibrinogen alpha chain (FGA), inter-alpha-trypsin inhibitor heavy chain H4 (ITIH4) and complement component 3. In the subsequent LC-MS/MS quantitation analysis, the levels of the three peptides (FGA-1033.4, ITIH4-2026.9, ITIH4-2051.1) exhibited a significant difference between the PE-positive and PE-negative groups. Further, the three-peptide panel yielded an area under the ROC curve (AUC) of 0.985 [95% confidence interval (CI) 0.965–1.000] and 0.923 (95% CI 0.845–1.000) in the discovery and validation cohorts respectively, with negative predictive values of 98.1–98.8% and positive predictive values of 73.1–85.3% that were much improved when compared with that of soluble fms-like tyrosine kinase-1/placental growth factor (sFlt-1/PlGF) ratio.

**Conclusions:**

We have discovered and validated a novel three-peptide biomarker panel predictive for the occurrence PE in pregnant women.

## Introduction

Preeclampsia (PE) is a pregnancy associated complication characterized by high blood pressure and proteinuria after 20 weeks of gestation and accompanied by multiple organ damage, such as heart, brain, and kidney (1, 2). PE related complications include but not limited to high risks of iatrogenic preterm delivery, intrauterine growth restriction, placental abruption, and perinatal mortality, along with maternal morbidity and mortality (3, 4). Currently, there is no reliable early warning indicators for PE due to a lack of effective diagnostic testing method, which poses a serious threat to the health of pregnant women and infants.

Extensive efforts have been invested in the search of PE predictive biomarkers. Some of the previous studies have focused on the risk assessment in the first trimester of pregnancy (5). However, the PE predictive models in early pregnancy, which often combines maternal background risk factors, imaging tests and serum biomarkers to increase sensitivity, displayed poor positive predictive values (PPV, 8–33%) in general population in which the prevalence of PE is low (6). False-positive patients who did not develop PE may have undergone unnecessary tests and prophylactic interventions with little benefit. Other investigations of PE predictive biomarkers have focused on suspected patients at a later stage of pregnancy (>20 gestational weeks) presenting with PE associated symptoms and/or unusual laboratory results (7, 8). For instance, the soluble fms-like tyrosine kinase-1 (sFlt-1) and placental growth factor (PlGF) has been proven to be effective in excluding PE with a negative predictive value (NPV) of 99.3%, although the PPV was still lower than 40% (7). Similar observation was made with renal function tests such as uric acid and cystatin C (9).

Peptidomics, a comprehensive analysis of native peptides using high performance liquid chromatography (HPLC) coupled with mass spectrometry (MS), is a powerful technology for unbiased screening of biomarkers of human diseases (10). Unlike proteomics, peptidomics focuses on the analysis of naturally occurring and endogenous low molecular weight (LMW) peptides and proteolytic fragments (MW < 10 kDa) which are considered as pathophysiological surrogates in signaling, proteolytic, and anti-proteolytic pathways in systemic diseases such as PE (11). For instance, using peptidomics, Wen et al. (11) identified the degradation patterns of serum specific protein throughout the progression of PE and discovered a 19-peptide panel that could be potentially used in PE prediction and differential diagnosis. Furthermore, despite a small cohort (*n* = 6, 3 PE and 3 controls), Dai et al. (12) found that the differentially expressed peptides were engaged in enzyme regulator activity, biological regulation, and coagulation cascades during pathological changes of PE. However, these previous peptidomic studies of PE prediction were hampered by their retrospective study design in nature and a lack of peptide quantitative validation in independent cohorts.

In this work, we have carried out a prospective peptidomic analysis in the suspected patients during PE development and progression. An analytical workflow of matrix-assisted laser desorption ionization-time of flight (MALDI-TOF) combined with high-resolution mass spectrometry was applied to identify differentially expressed peptides. The selected peptide candidate biomarkers were further verified and validated using an in-house developed liquid chromatography-tandem mass spectrometry (LC-MS/MS) quantitative method in two independent cohorts.

## Materials and methods

### Subjects and sample collection

The enrollment criteria for women with suspected PE are described as follows (9). The recruited singleton pregnant women were at least 18 years old and between 20 and 36 gestational weeks (GWs). In addition, one of the following recruiting criteria had to be met for patient recruitment: new onset of elevated blood pressure (BP) (systolic BP >120 and <160 mmHg and/or diastolic BP >80 and <110 mmHg) or proteinuria (≥2+ by dipstick); aggravation of preexisting hypertension or proteinuria; or persistent symptoms of upper abdominal pain, edema, headache, visual impairment, abnormal weight gain (>1 kg/week), decreased platelets (<150^*^109/L), elevated liver transaminase (alanine transferase >55 U/L or aspartate transaminase >34 U/L), fetal growth restriction (estimated fetal weight or abdominal circumference <10th percentile according to the charts routinely used by Obstetric Department at our institute), increased pulsatility index (PI) of the uterine artery (PI > 0.878), abnormal uterine ultrasound perfusion during mid-pregnancy, or uterine artery flow notching. The exclusion criteria included: confirmed diagnosis of PE or Hemolysis Elevated Liver enzymes and Low Platelets (HELLP) syndrome at enrollment. The recruited pregnant subjects had their sera collected at their first visits with onset of the suspected symptoms, and followed up for the presence (“PE-positive” group) or absence (“PE-negative” group) of preeclampsia until delivery.

The preeclampsia diagnosis was determined with the diagnostic criteria proposed by the 2019 ACOG Practice Bulletin (6), in which preeclampsia was defined as gestational hypertension (systolic/diastolic blood pressure ≥140/90 mmHg) in previously normotensive women accompanied by proteinuria (urine protein ≥300 mg/24 h) or end-organ damage after 20 weeks of gestation. The methods for serum levels of sFlt-1 and PlGF were listed in the Supplementary Methods.

### Biomarker study design

**Figure 1:**
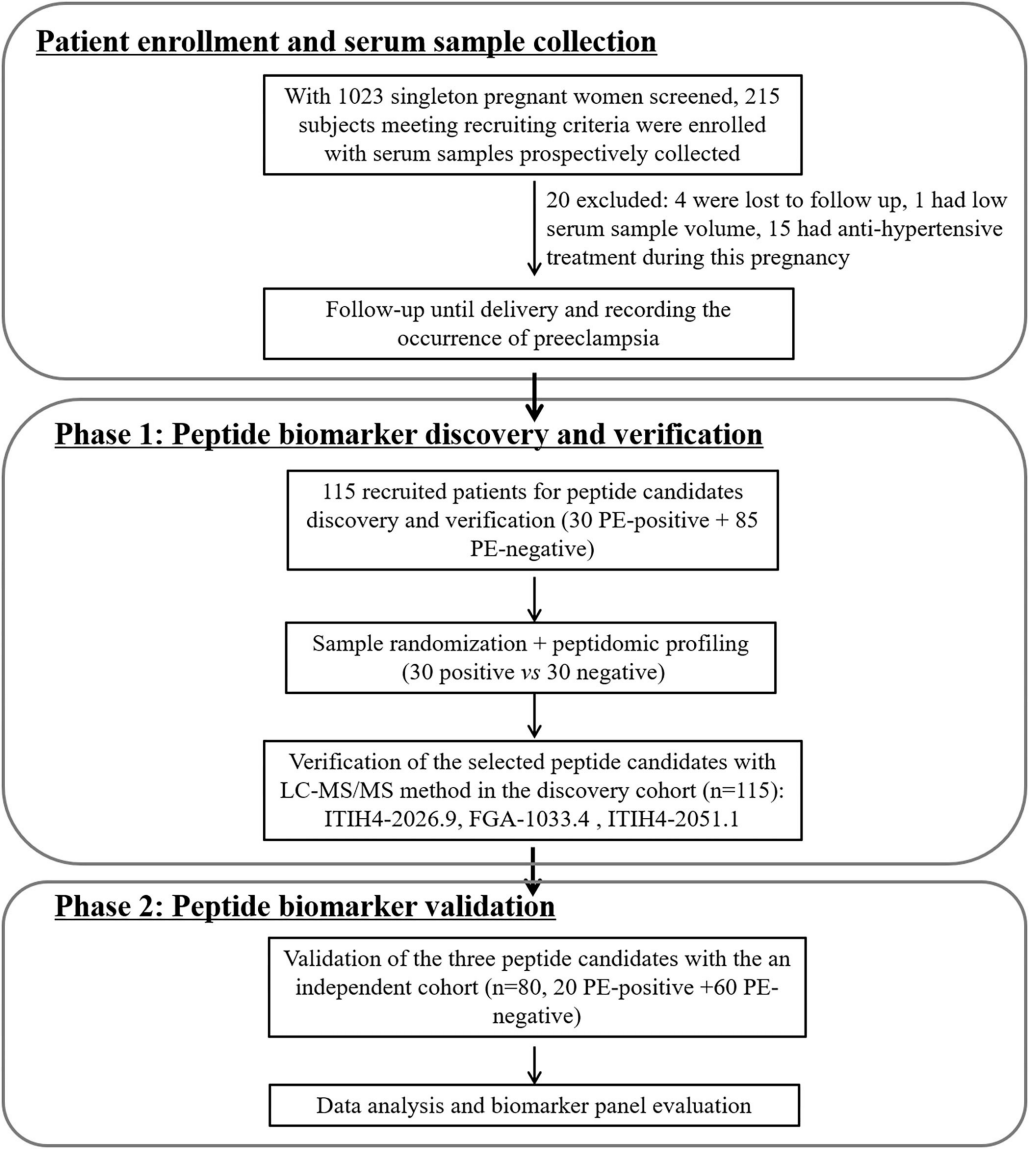
Schematic diagram for patient enrollment and peptide biomarker candidates discovery and validation for preeclampsia prediction.

The study design of biomarker development followed the principles of the PRoBE (prospective-specimen collection, retrospective-blinded-evaluation) (13), in which all the serum samples were prospectively collected from the enrolled patients before PE development. As depicted in Figure 1, with 1,023 singleton pregnant women screened by the clinician team, totally 215 subjects meeting recruiting criteria were initially enrolled. Twenty of them were excluded due to incomplete follow-up (*n* = 4), low serum sample volume (*n* = 1) or receiving anti-hypertensive treatment during pregnancy (*n* = 15). For peptidomic analysis, peptide biomarker discovery and verification, 115 pregnant women (30 PE-positive and 85 PE-negative) enrolled from January 2018 to August 2018 were included. For the peptide candidates validation, an independent cohort of 80 subjects (20 PE-positive and 60 PE-negative, recruited from Oct 2018 to Jan 2019) was used. The schematic diagram of the study designed for the two phases of serum peptide biomarker development for PE prediction was shown in Figure 1.

### Mass spectrometry-based serum peptidomics and peptide candidates quantitation

The method details for serum pretreatment, peptidomic profiling, data processing (14, 15), peptide candidates identification (16) and quantitation by liquid chromatography tandem mass spectrometry (LC-MS/MS) (17) were summarized in the Supplementary Methods. In the peptide quantitation, the details for calibration curve setup, internal standard spiking concentration and mass spectrometry parameter setting were included in Supplementary Table 1.

### Statistical analysis

Data analysis was performed using statistical software SPSS 23.0. The Kolmogorov–Smirnov test was used to evaluate the normality of the data distribution. Numerical values were expressed as the mean and standard deviation (SD) for variables with normal distribution and as the median and percentiles for non-normally distributed data. Comparisons between the two groups were performed using the *t*-test (for normal distribution) or Mann-Whitney-*U* test (for non-normal distribution). Categorical variables were expressed as frequencies and proportion; comparisons between the two groups were tested by Chi-square test. The receiver operating characteristics (ROC) curve was used to analyze the predictive values of the markers for preeclampsia. Multivariate logistic regression analysis was applied to obtain the combined ROC curve. Sensitivity, specificity and cut-off values determined by the Youden's index were reported. The correlation analysis adopted in present study was Kendall's correlation method.

## Results

### Discovery and identification of differently expressed peptides between PE-positive and PE negative groups

Following the PRoBE design and the enrollment criteria, totally 195 suspected subjects with PE-relevant clinical symptoms or abnormal laboratory results had their serum sample collected right upon recognition by our clinical team. The demographic information for the recruited patients, including age, pre-pregnancy BMI, gravidity, parity, and sampling gestation week, were summarized in Table 1. Supplementary Tables 2, 3 listed detailed information of each participant in the discovery cohort (*n* = 115) and the validation cohort (*n* = 80) respectively. As shown in Table 1, for any of the listed subjects' demographic factors, there was no significant difference between the PE-posiive and PE-negative groups.

**Table 1:** Demographic information for the discovery cohort and the validation cohort.

	**Age**	**Pre-pregnancy BMI**	**Gravidity (** ***n*** **, %)**			**Parity (** ***n*** **, %)**			**Sampling week**
			**1**	**2**	**≥3**	**0**	**1**	**2**	
**Discovery cohort (*****n*** **=** **115)**									
PE-positive (*n* = 30)	33.4 ± 4.0	25.4 ± 4.4	10 (33.3%)	10 (33.3%)	10 (33.3%)	20 (66.7%)	9 (30.0%)	1 (3.3%)	28.4 ± 4.4
PE-negative subgroup (*n* = 30)	31.0 ± 4.2	24.0 ± 3.8	12 (42.9%)	9 (32.1%)	7 (25.0%)	20 (71.4%)	8 (28.6%)	0 (0.0%)	27.6 ± 5.0
PE-negative total (*n* = 85)	32.9 ± 4.4	24.2 ± 4.3	34 (43.0%)	24 (30.4%)	21 (26.6%)	54 (68.4%)	23 (29.1%)	2 (2.5%)	28.9 ± 5.3
*p* ^a^	0.120	0.222		0.706			0.609		0.528
*p* ^b^	0.640	0.217		0.632			0.968		0.561
**Validation cohort (*****n*** **=** **80)**									
PE-positive (*n* = 20)	32.6 ± 5.6	24.5 ± 4.2	8 (40.0%)	5 (25.0%)	7 (35.0%)	13 (65.0%)	7 (35.0%)	0 (0.0%)	29.6 ± 3.1
PE-negative (*n* = 60)	33.8 ± 4.4	25.1 ± 4.6	19 (35.8%)	19 (35.8%)	15 (28.3%)	33 (62.3%)	19 (35.8%)	1 (1.9%)	28.3 ± 4.6
*p* ^c^	0.310	0.619		0.669			0.820		0.149

a Comparison between PE-positive (*n* = 30) and PE-negative subgroup (*n* = 30) of the discovery cohort;

b comparison between PE-positive (*n* = 30) and PE-negative (*n* = 85) of the discovery cohort;

c comparison between PE-positive (*n* = 20) and PE-negative (*n* = 60) of the validation cohort.

As depicted in Figure 1, the peptide biomarker development consisted of 2 phases: discovery and validation. In the discovery group, serum samples from all PE-positive subjects (*n* = 30) and 30 PE-negative patients with matching demographic information were used for the peptide discovery study. A total of 117 informative peaks were detected using the MALDI-TOF peptidomic analytical platform described above. Thirty-two out of the 117 features were significantly different between the PE-positive and PE-negative groups (*p* < 0.05), with a fold-change of ≥2.00 or ≤0.40 and an average peak intensity of ≥100 in at least one group (Table 2, Supplementary Table 4).

**Table 2:** Significantly differentially expressed mass peaks with peptide identification by LTQ-Orbitrap-MS.

**Mass, *m*/*z*^a^**	**FDR-adjusted *p*-value**	**PE-positive (*****n*** = **30)**		**PE-negative (*****n*** = **30)**		**FC^c^**	**Peptide ID and sequence**
		**Mean**	**SD^b^**	**Mean**	**SD**		
2026.94	1.02E-09	284.3	171.3	19.3	8.5	14.76	ITIH4: QLGLPGPPDVPDHAAYHPF
1876.85	1.33E-09	105.7	65.1	16.9	10.3	6.24	Complement C3: YSIITPNILRLESEET
3276.45	1.30E-09	152.9	59.9	34.6	22.6	4.42	FGA: SSSYSKQFTSSTSYNRGDSTFESKSYKM (+15.99)A^d^
3260.46	1.23E-09	1117.7	419.9	270.3	164.9	4.14	FGA: SSSYSKQFTSSTSYNRGDSTFESKSYKMA
2192.10	1.56E-03	113.2	103.1	45.6	32.8	2.48	Complement C3: SPMYSIITPNILRLESEET
1033.40	1.62E-03	110.2	63.9	46.2	31.1	2.38	FGA: SSSYSKQFT
2051.08	5.75E-04	436.8	330.0	218.4	260.1	2.00	ITIH4: YYLQGAKIPKPEASFSPR
5900.70	1.56E-04	430.0	282.0	1065.5	718.6	0.40	FGA: SSSYSKQFTSTSYNRGDSTFESKSYKMADEAGS-EADHEGTHSTKRGHAKSRPV

a Mass determined by LTQ-Orbitrap-MS;

b standard deviation;

c fold change;

d (+15.99) indicating oxidation on the methylsulfinyl group of methionine residue. ITIH4, Inter-alpha-trypsin inhibitor heavy chain H4; FGA, Fibrinogen alpha chain.

Using LTQ-Orbitrap-MS analysis, 8 of 32 differentially expressed peptides were identified by matching the Sequest™ database (Table 2). With predicted peptide sequences listed in Table 2, the peptides 1,033.40 *m/z*, 3,260.46 *m/z*, 3,276.45 *m/z*, 5,900.70 *m/z* were identified to be fibrinogen alpha chain (FGA); the peptides 2,026.94 *m/z*, 2,051.08 *m/z* were identified to be Inter-alpha-trypsin inhibitor heavy chain H4 (ITIH4); the peptides 1,876.85 *m/z*, 2,192.10 *m/z* were identified to be complement component 3 (C3). Considering the redundancy of the identified peptide sequences and convenience of peptide synthesis, the following four peptides were carried forward for the next absolute quantitation step by LC-MS/MS: FGA-1033.4, ITIH4-2026.9, ITIH4-2051.1, C3-1876.9.

### Peptide biomarker quantitation by LC-MS/MS and sFlt-1/PlGF measurements

The selection of precursor and product ion of each peptide candidate was performed by direct infusion of the synthesized standard peptides. The resulting ion transition pairs chosen, calibrator and MS parameter setting were listed (Supplementary Table 1). As seen in Supplementary Figure 1, the identical peaks were observed in both calibrators and pooled patient samples for all the target peptides analyzed except C3-1876.9. As shown in Supplementary Figure 1, no corresponding peak for C3-1876.9 was observed in the pooled patient serum, suggesting that C3-1876.9 identified by high-resolution MS was a pseudo-target which was not further investigated in the following experiments. All of the calibration curves were linear within the concentration ranges set for each of the peptides (*r* > 0.995) (Supplementary Figure 2). The original peptide quantitation data using the in-house established LC-MS/MS method and the measurements of serum levels of sFlt-1/PlGF in both discovery and validation cohorts were recorded in Supplementary Tables 2, 3.

### Performance of the peptide biomarker candidates and sFlt-1/PlGF ratio in PE prediction

**Figure 2:**
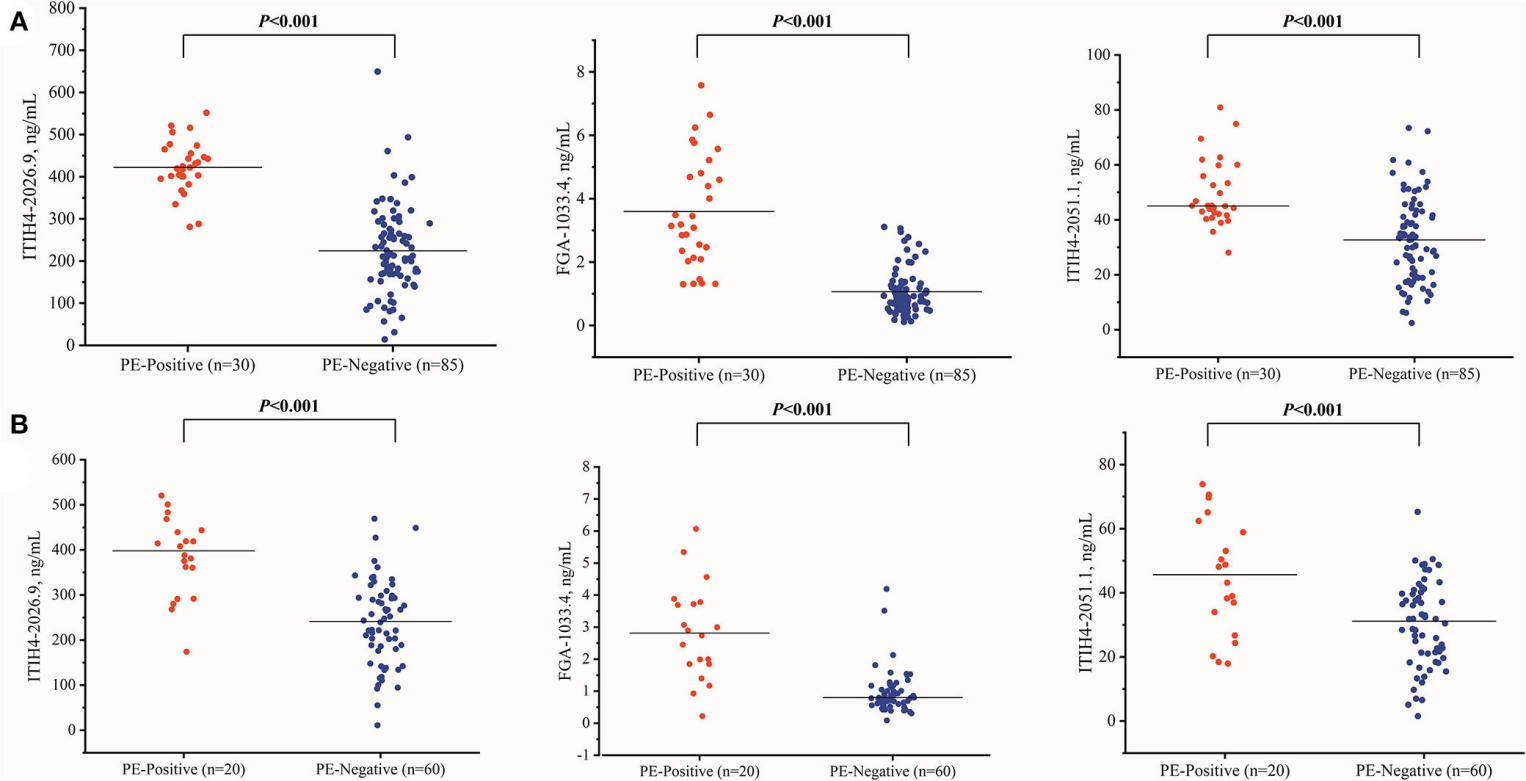
The three peptide candidates quantitation of the PE-positive and PE-negative groups in the discovery **(A)** (PE-positive *n* = 30, PE negative *n* = 85) and validation **(B)** (PE-positive *n* = 20, PE negative *n* = 60) cohorts.

**Figure 3:**
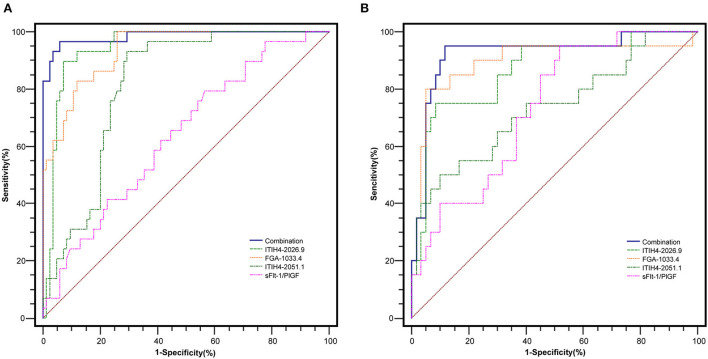
The ROC analysis of the peptide candidates for PE prediction in the discovery **(A)** (PE-positive *n* = 30, PE negative *n* = 85) and validation **(B)** (PE-positive *n* = 20, PE negative *n* = 60) cohorts.

With the similar changing pattern seen in the peptidomic study by MALDI-TOF, all three peptides, including FGA-1033.4, ITIH4-2026.9, ITIH4-2051.1, were found significantly elevated (*p* < 0.001) in the PE-positive group in the discovery cohort (*n* = 115) (Supplementary Table 5, Figure 2). Interestingly, a three-dimensional (3-D) scattered plot also showed almost complete separation of the PE-positive and PE-negative patients, suggesting their potential discriminating power when used in a combinational panel (Supplementary Figure 3). Moreover, in the ROC analysis with the discovery cohort, the area under curves (AUCs) of the three candidate peptides and sFlt-1/PlGF ratio were listed in the order of decreasing values: 0.985 (95% CI, 0.965–1.000) (three peptides combined), 0.946 (95% CI, 0.905–0.988) (ITIH4-2026.9), 0.939 (95% CI, 0.898–0.980) (FGA-1033.4), 0.820 (95% CI, 0.744–0.896) (ITIH4-2051.1), 0.637 (95% CI, 0.525–0.750) (sFlt-1/PlGF). The predictive panel containing 3 peptides showed higher accuracy in PE prediction than any of the peptide biomarker along (*p* < 0.05) (Figure 3), and achieved a sensitivity of 96.7%, a specificity of 94.1%, PPV of 85.3%, and NPV of 98.8% at the cutoff determined by the Youden's index (Table 3).

**Table 3:** ROC analysis of the sFlt-1/PlGF and peptide biomarker candidates in preeclampsia prediction.

	**sFlt-1/PlGF**	**ITIH4-2026.9**	**FGA-1033.4**	**ITIH4-2051.1**	**Combined^a^**
**Discovery cohort (*****n*** **=** **115)**					
AUC (95% CI)	0.637 (0.525–0.750)	0.946 (0.905–0.988)	0.939 (0.898–0.980)	0.820 (0.744–0.896)	0.985 (0.965–1.000)
Sensitivity, %	79.3	90.0	100.0	93.3	96.7
Specificity, %	43.5	92.9	74.1	70.6	94.1
Positive predictive value, %	32.4	81.8	57.7	52.8	85.3
Negative predictive value, %	86.0	96.3	100.0	96.8	98.8
*p-*value of AUCs comparison	<0.001	0.036^b^	0.026^c^	<0.001^d^	–
**Validation cohort (*****n*** **=** **80)**					
AUC (95% CI)	0.733 (0.617–0.850)	0.866 (0.771–0.961)	0.896 (0.795–0.997)	0.734 (0.598–0.871)	0.923 (0.845–1.000)
Sensitivity, %	95.0	75.0	80.0	50.0	95.0
Specificity, %	48.3	91.7	95.0	90.0	88.3
Positive predictive value, %	38.0	75.0	84.2	62.5	73.1
Negative predictive value, %	96.7	91.7	93.4	84.4	98.1
*p-*value of AUCs comparison	0.004	0.040^b^	0.185^c^	0.002^d^	–

a The three-peptide combined panel in ROC analysis;

b AUCs comparison between ITIH4-2026.9 and three-peptide panel;

c AUCs comparison between FGA-1033.4 and three-peptide panel;

d AUCs comparison between ITIH4-2051.1 and three-peptide panel. ROC, receiver operating characteristic; AUC, area under curve; CI, confidence interval.

In the independent validation cohort, the three peptides exhibited significantly higher levels in the PE-positive group in comparison to the PE-negative group (Supplementary Table 5, Figure 2). Similar separation pattern was observed between the PE-positive and PE-negative patients in the 3-D scatter plot (Supplementary Figure 3). In the ROC analysis, the FGA-1033.4 showed the best predictive power with an AUC of 0.896 (95% CI, 0.795–0.997), followed by ITIH4-2026.9 (AUC = 0.866, with 95% CI of 0.771–0.961), ITIH4-2051.1 (AUC = 0.734, with 95% CI of 0.598–0.871) and sFlt-1/PlGF (AUC = 0.733, with 95% CI of 0.617–0.850). The three-peptide panel yielded a better AUC (0.923, with 95% CI of 0.845–1.000) than that of sFlt-1/PlGF, ITIH4-2051.1 or ITIH4-2026.9, but not FGA-1033.4. With the cut-off determined with Youden's Index, the three-peptide panel showed a sensitivity of 95.0%, a specificity of 88.3%, PPV of 73.1%, and NPV of 98.1% (Table 3). As shown in Table 4, interestingly, all of the three peptides showed slightly negative correlation with serum PlGF and marginally positively correlation with sFlt-1/PlGF ratio. No significant correlation was observed between any of the three peptide candidates and the rest of parameters included, such as sFlt-1, age, pre-pregnancy BMI, gravidity and parity.

**Table 4:** Peptide candidates' correlation analysis with clinical markers.

	**sFlt-1**	**PlGF**	**sFlt-1/PlGF**	**Age**	**Pre-pregnancy BMI**	**Gravidity**	**Parity**
ITIH4-2026.9, *r*^d^	0.101	−0.105	0.136	0.068	0.035	0.102	0.124
FGA-1033.4, *r*^d^	0.011	−0.120	0.084	0.000	−0.005	0.094	0.040
ITIH4-2051.1, *r*^d^	0.055	−0.116	0.109	0.027	−0.003	0.108	0.143
*p* ^a^	0.054	0.045	0.010	0.211	0.502	0.082	0.051
*p* ^b^	0.828	0.023	0.112	0.995	0.928	0.111	0.530
*P* ^c^	0.297	0.027	0.037	0.613	0.955	0.066	0.024

a Comparison between ITIH4-2026.9 and other clinical markers;

b comparison between FGA-1033.4 and other clinical markers;

c comparison between ITIH4-2051.1 and other clinical markers;

d *r* stands for the calculated correlation coefficient with the Kendall's method.

## Discussion

Using the state-of-the-art MALDI-TOF coupled with Linear Trap Quadropole Orbitrap mass spectrometry, our study discovered a novel three-peptide panel which has exhibited promising performance in the prediction of PE occurrence in pregnant women. The finding was further validated in an independent cohort with comparable accuracy, sensitivity and specificity. Furthermore, this three-peptide model yielded a significantly improved PPV of 73.1–85.3% compared with that of sFlt-1/PlGF ratio as predictive markers (32.4–38.0%), which was close to what was observed in our previous study (9). Therefore, our panel is more accurate in PE prediction with suspected patients and offers a feasible and efficient strategy for “rule-in” and “rule-out” high risk patients who may need extra clinical care.

MALDI-TOF-MS is a cutting-edge technology that offers sensitive and accurate identification of proteomic biomarkers of disease condition (18). The bead-based fractionation method, which selectively separates peptides according to different chemical chromatographic surfaces on the outer layer of magnetic beads, has been developed for direct use in MALDI-TOF-MS analysis (19). In combination with the bioinformatics BE Software™ (Bioyong Tech., Beijing, China), weak cation exchange magnetic beads (WCX-MB) pretreatment and MALDI-TOF-MS analysis provides a powerful tool for analyzing and identifying novel biologically informative molecules and has been successfully applied to biomarker research (20). In this work, the MALDI-TOF peptidomic analysis in combination with a high-resolution mass spectrometry identified 8 differentially expressed peptide candidates belonging to three proteins: FGA, ITIH4 and C3 (Table 2). However, the peptideC3-1876.9 was not found in the pooled serum of pregnant women by the LC-MS/MS method. This apparent discrepancy may be explained by the different serum pretreatment steps applied in the MALDI/QE-based peptidomic analysis vs. the LC-MS/MS peptide quantitation.

Intriguingly, according to an earlier retrospective peptidomic study, 13 peptides from FGA and 1 peptide from ITIH4 (different peptide sequences compared with our peptide candidates) were found remarkably upregulated in PE, suggesting the pathological relevance of the two proteins in PE disease progression (11). Similar observation was made in our peptidomic study in which 4 peptides from FGA and 2 peptides from ITIH4 were differentially expressed and identified, suggesting there is a disease-specific proteolytic degradation pattern of the parent proteins. The fact that multiple peptides from the same proteins were identified further confirmed the accuracy and reliability of our discovery.

FGA and ITIH4 are derived from proteins known to be involved in the pathophysiology of PE in acute inflammatory and defense response. FGA is encoded by the human FGA gene, which is a component of fibrinogen that consists of pairs of three different polypeptide chains, including the α, β and γ chains joined by disulfide bonds to form a symmetric dimeric structure (21). Fibrinogen is involved in blood clotting, but has also been implicated as an inflammatory mediator in several diseases, including rheumatoid arthritis (RA), multiple sclerosis, and Alzheimer's disease (22). The up-regulation of FGA is believed to contribute to the pathogenesis of preeclampsia by participating in the trophoblast recast of uterine spiral artery, activation of systemic inflammatory response and injury of endothelial cells (23). Furthermore, FGA was found to be an independent risk factor associated with increased cardiovascular morbidity and mortality in PE patients (24).

Inter-alpha-trypsin inhibitor heavy chain H4 (ITIH4) refer to the heavy chains of protein members belonging to the ITI family, which is involved in stabilization of the extracellular matrix (25). ITIH4 is a 120-kDa serum glycoprotein secreted primarily by liver and is associated with inflammation and carcinogenesis. As an acute phase response protein, ITIH4 is increased in response to infection and inflammation, and may provide important diagnostic information during surgical trauma (26). In previous studies, it has been indicated that ITIH4 was significantly up-regulated in serum samples of patients with ovarian, breast or bladder cancers (27). Interestingly, in pigs, endometrial gene expression of a 30-kDa fragment of ITI-H4 was detected during the estrous cycle and early pregnancy. Geisert and his colleagues suggested that the ITIH4 may be expressed to protect the maternal uterus as an acute phase protein during primary pregnancy (28). Further, ITIH4 is believed to contribute the pathogenesis of preeclampsia through excessive activation of inflammatory immunity, leading to disturbance of maternal-fetal immune balance and resulting in “shallow implantation of the placenta”. Another previous study showed that overexpression of the 36-kDa fragmented form ITIH4 may induce a strong inflammatory response in pregnant women, and consequently the pregnancy may fail (29). However, the exact biological function of ITIH4 in PE is still not fully understood.

While a large number of studies have focused on preeclampsia prediction during pregnancy, very few serum predictive markers were successfully implemented in clinical practice mainly due to low accuracy. With the low prevalence of preeclampsia in the general pregnant population, it would be economically inefficient to universally apply laboratory biomarker test(s) during pregnancy. In the publication of evaluating sFlt-1/PlGF ratio in PE prediction by Zeisler et al. (7), the authors narrowed down the targeting patients who presented with PE-related clinical and/or laboratory abnormalities. A similar patient recruiting strategy was adopted in our study. With a straight focus on the subgroup of suspected patients with relevant symptoms, it allows medical resources to be better targeted on the patients that are more likely to develop preeclampsia.

In our study, the three peptides FGA-1033.4, ITIH4-2026.9, ITIH4-2051.1 were found to be able to accurately predict the occurrence of PE in middle or late pregnancy. However, the exact biological roles of FGA and ITIH4 in PE development are still largely unknown and need to be further investigated. In addition, with the help of the peptide quantitation method developed and validated in present study, the cut-off values of the three peptides should be determined in a large and multi-center study for future clinical application.

This work is a well-designed prospective study, with rigorous technical design of discovery and validation studies using state-of-the-art MS in independent patient cohorts. However, a few limitations still exist. First, all the participants included in our study were Chinese; the performance of this peptide panel in diverse ethnic backgrounds especially in Western countries needs to be further tested. Second, the overall size of the cohorts was relatively small and all the patients were from a single site.

## Conclusions

Our study is the first to develop and validate a predictive panel consisting of three circulating peptides that can effectively rule-out or rule-in PE development in the suspected patients. The changes in these peptides predated the onset of the disease and were present in both of the discovery and validation cohorts. With the implement of a straightforward LC-MS/MS quantitation method, these circulating peptides may provide information on PE development and act as potential biomarkers for PE prediction in clinical practice. However, the pathological mechanism in which the identified peptide markers may participate requires further investigation.

## Data availability statement

The datasets presented in this study can be found in online repositories. The names of the repository/repositories and accession number(s) can be found below: https://www.iprox.cn/page/project.html?id=IPX0004482000.

## Ethics statement

This study was approved on 07 June 2021, by the Ethics Committee of Beijing Obstetrics and Gynecology Hospital, Capital Medical University (approval number: 2021-KY-059-01). Written informed consent for participation was not required for this study in accordance with the national legislation and the institutional requirements.

## Author contributions

SZ: conceptualization, data curation, and writing—original draft. CY: project administration and supervision. YZha: conceptualization, data curation, and funding acquisition. ZJ and SS: investigation and methodology. YLu and LM: investigation. CL, XianL, and YC: methodology. YLi, YLiu, LC, JW, and ZX: data curation. YZhe: data curation and investigation. ZS, RL, XY, and HY: writing—review and editing. XiaoL: conceptualization, data curation, and project administration. ZZ: conceptualization, supervision, and writing—review and editing. ZC: conceptualization, supervision, funding acquisition, and writing—review and editing. All authors contributed to the article and approved the submitted version.

## Funding

This work was supported by the Capital Medical University (No. PYZ20056) and the Beijing Municipal Administration of Hospitals Incubating Program (No. PX2020060). The funding bodies did not take part in the design of the study, the collection, analysis and interpretation of the data, or manuscript writing.

## Conflict of interest

Authors CL, XianL, and YC were employed by company SCIEX. The remaining authors declare that the research was conducted in the absence of any commercial or financial relationships that could be construed as a potential conflict of interest.

## Publisher's note

All claims expressed in this article are solely those of the authors and do not necessarily represent those of their affiliated organizations, or those of the publisher, the editors and the reviewers. Any product that may be evaluated in this article, or claim that may be made by its manufacturer, is not guaranteed or endorsed by the publisher.
