# Supplementary material for: Serum peptidomic screening identified circulating peptide biomarkers predictive for preeclampsia
Source: Front Cardiovasc Med. 2022 Oct 11;9:946433. doi: 10.3389/fcvm.2022.946433 (PMC9595599; doi:10.3389/fcvm.2022.946433)
Supplement: Supplementary file 1 [file Data_Sheet_1.docx]

**Supplementary Methods**

**Measurement of the serum levels of sFlt-1 and PlGF**

The maternal levels of sFlt-1 (Cat No. YZB/GER5424-2014, Germany, Roche Diagnostics) and PlGF (Cat No. YZB/GER5425-2014, Germany, Roche Diagnostics) were measured on the fully automated electrochemiluminescence immunoassay platform COBAS e411 (Germany, Roche Diagnostics).

**Serum pretreatment, peptidomic profiling and data processing in the peptidomic study**

The maternal blood from each participant (3 ml) was drawn when they were enrolled and left to clot for 30 min followed by centrifugation for 10 min at 2300 x g. The serum aliquots (1 ml) were separated and stored at -80℃ until being tested.

All the serum samples were subjected to fractionation using the weak cation exchange magnetic bead (SPE-C kit, Bioyong Tech., Beijing, China) designed for low-molecular-weight proteins or peptide isolation as described previously^[1.2]^. According to the manufacturer’s instructions, after binding, washing and eluting steps, the enriched proteins/peptides were ready for anchor-chip spotting and peptide profiling. The peptidomic profiling was performed with a linear MALDI-TOF mass spectrometer (Clin-TOF-II, Bioyong Tech., Beijing, China), with a mass calibration range of 1000-10000 Da. Three standard peptides (molecular weights of 1532.8582 Da, 2464.1989 Da, and 5729.6087 Da, Product Numbers P2613, A8346, and I6279, respectively; Sigma) were used as external quality controls for ensure the molecular weight (MW) deviation was within 100 ppm. For data processing, each spectrum was normalized, baseline-corrected and smooth-applied using default parameters of the BioExplorer software (Bioyong Tech., Beijing, China). All the quality peaks with a signal-to-noise (S/N) ratio greater than 5 were obtained. Welch's T test and Wilcoxon-Mann Whitney U test were conducted for the peak signal comparison between the two patient groups according to the distribution of the data. In order to decrease the false discovery rate (FDR) for biomarker selection, local FDR based on *p* value was calculated to adjust the multiple comparisons (*p*<0.05 considered as statistically significant) between different groups.

**Identification of peptides biomarkers for PE prediction**

The serum peptide identification was performed by analyzing the pooled serum samples that had been pretreated with the SPE-C kit, with a nano-liquid chromatography-electrospray ionization-tandem mass spectrometry (nano-LC/ESI-MS/MS) system consisting of an Aquity UPLC platform (Waters, MA, USA) and a Linear Trap Quadropole (LTQ) Orbitrap XL mass spectrometer equipped with a nano-ESI source (Thermo Fisher, MA, USA). As previously stated^[1,2]^, the processed peptide solutions were loaded to a C18 trap column followed by a C18 analytical column (nanoACQUITY) at a flow rate of 400 nl/min with mobile phases A (5% acetonitrile, 0.1% formic acid) and B (95% acetonitrile, 0.1% formic acid) that were used for the gradient elution. The MS instrument was operated in a data-dependent model, with similar Orbitrap MS parameter setup as detailed in Lin’s study^[3]^. The obtained chromatograms from the MS/MS protocol were analyzed with BioworksBrowser^TM^ 3.3.1 SP1, and the resulting mass lists were searched for in the Sequest™ (IPI Human (3.45)) database. The parameters for generating the peak list were set at 50 ppm and 1 Da for the parent ion and fragment mass, respectively.

**Peptides quantitation by liquid chromatography tandem mass spectrometry (LC-MS/MS)**

With the peptidomic analysis described above, four peptides were selected for further quantitation: QLGLPGPPDVPDHAAYHPF (ITIH4-2026.9), SSSYSKQFT (FGA-1033.4), YYLQGAKIPKPEASFSPR (ITIH4-2051.1) and YSIITPNILRLESEET (C3-1876.9), in which underlined amino acids were isotopically labeled in the corresponding heavy peptides (^13^C/^15^N labeling). All the standard light (unlabeled) and heavy peptides (labeled) were synthesized, with purity determined by HPLC and amino acid analysis (Synpeptide, Najing, China). The lyophilized peptide stocks were initially solubilized in a 0.1% formic acid water solution, with the concentration of 1 mg/ml.

As described previously^[4]^, to circumvent the issue of existing endogenous peptides in human serum, the reverse calibration strategy was adopted in constructing the standard curves. Basically, in the pooled clinical serum samples, a constant amount of light peptide was spiked on top of the endogenous peptide to create an internal standard and varying amounts of heavy peptide were spiked to create a set of concentration standards. The clinical samples to be tested were spiked with the same amounts of heavy peptides as internal standards spiked into the matrix used to create reverse calibration curves, in which the light peptides were used as internal standards. A 7-point external calibration curve was constructed for each of the three peptides, with concentration ranges included in parentheses: ITIH4-2026.9 (2.5-500 ng/ml), FGA-1033.4 (0.25-50 ng/ml), ITIH4-2051.1 (2.5-500 ng/ml). Details for calibration curve setup, internal standard spiking concentration and mass spectrometry parameter setting were included in Supplementary Table 1. As the presence of the C3-1876.9 peptide was not confirmed in the pooled patient serum by the LC-MS/MS method, this peptide was not included in the subsequent peptide quantitation analysis.

For peptide preparation, 100 μl serum sample or calibrator was mixed with 300 μl 25% acetonitrile (ACN) followed by sedimentation at 4℃ for 30 min; another 740 μl 100% ACN was added to the mixture to allow complete protein precipitation, followed by centrifugation at 6000 x g for 30 min. The supernatant was transferred to a fresh tube and evaporated to dryness under nitrogen and reconstituted with 100 μl 0.1% formic acid water solution. The reconstitute was further centrifuged at 10000 x g for 100 min, after which 45 μl supernatant mixed with 5 μl internal stand concentrate solution was prepared and 10 μl injection volume was used for LC-MS/MS analysis.

The LC-MS/MS analysis was performed using an AB Sciex 5500 mass spectrometer coupled with a Shimadzu Nexera X2 HPLC system. A Waters BEH C18 column (2.1 x 100 mm, 1.7 µm) was used and maintained at a constant temperature of 40℃ during operation. The mobile phase A was composed of 98% water, 2% ACN, 0.1% formic acid; the mobile phase B was composed of 98% ACN, 2% water, 0.1% formic acid. The chromatography gradient conditions (%B) were set as follows (for a total run time of 6 min) with a follow rate of 0.2 ml/min: 10% B for 0-0.8 min, 10%-35% B for 0.8-2.5 min, 35%-90% B for 2.5-3.3 min, 90% B for 3.3-4.3 min, 90%-5% B for 4.3-4.4 min, 5% B for 4.4-6.0 min.

**Supplementary Tables**

**Supplementary Table 1** Peptide candidates quantitation instrumental setup by LC-MS/MS

|  |  |  |  |  |  |
| --- | --- | --- | --- | --- | --- |
| Peptide and sequence |  | MW | LC-MS/MS instrumental setting^a^ | | |
|  |  |  | Ion pair-1 (DP, CE, CXP)^b^ | Ion pair-2 (DP, CE, CXP)^b^ | Ion pair-3 (DP, CE, CXP)^b^ |
| ITIH4-2026.9: QLGLPGPPDVPDHAAYHPF | Light peptide | 2026.9 | 677.0/263.3 (45, 38, 8.2) | 677.0/299.3 (45, 35, 16) | 677.0/1054.5 (45, 35, 26) |
|  | Heavy peptide | 2033.9 | 679.2/263.2 (45, 36,15) | 679.2/299.3 (45, 36, 16) | 679.2/1054.5 (45, 36, 16) |
| FGA-1033.4: SSSYSKQFT | Light peptide | 1033.4 | 517.9/610.3 (100, 25, 20) | 517.9/768.4 (100, 24, 17) | 517.9/860.4 (100, 23, 18) |
|  | Heavy peptide | 1041.4 | 521.9/618.4 (100, 26, 20) | 521.9/766.4 (100, 24, 20) | 521.9/868.5 (100, 23, 20) |
| ITIH4-2051.1: YYLQGAKIPKPEASFSPR | Light peptide | 2051.1 | 684.9/327.2 (120, 37, 17) | 684.9/890.4 (120, 41, 25) | 684.9/1115.6 (120, 41, 26) |
|  | Heavy peptide | 2058.1 | 687.4/327.2 (120, 37, 19) | 687.4/890.4 (120, 41, 25) | 687.4/1115.6 (120, 41, 25) |
| C3-1876.8: YSIITPNILRLESEET | Light peptide | 1876.9 | 939.7/364.2 (120, 48, 19) | 939.7/251.1 (120, 52, 14) | 939.7/976.5 (120, 51, 14) |
|  | Heavy peptide | 1883.8 | 943.2/364.2 (120, 48, 20) | 943.2/251.1 (120, 52, 15) | 943.2/983.5 (120, 51, 17) |

**Supplementary Table 1** Peptide candidates quantitation instrumental setup by LC-MS/MS (continued)

|  |  |  |  |  |
| --- | --- | --- | --- | --- |
| Peptide and sequence |  | MW | Calibrators (ng/ml) | Internal standard |
|  |  |  |  |  |
| ITIH4-2026.9: QLGLPGPPDVPDHAAYHPF | Light peptide | 2026.9 | - | 50 ng/ml^c^ |
|  | Heavy peptide | 2033.9 | 10, 20, 50, 200, 500 | 50 ng/ml^d^ |
| FGA-1033.4: SSSYSKQFT | Light peptide | 1033.4 | - | 1 ng/ml^c^ |
|  | Heavy peptide | 1041.4 | 0.25, 0.5, 1, 2, 5, 10, 50 | 1 ng/ml^d^ |
| ITIH4-2051.1: YYLQGAKIPKPEASFSPR | Light peptide | 2051.1 | - | 10 ng/ml^c^ |
|  | Heavy peptide | 2058.1 | 2.5, 5, 10, 20, 50, 200, 500 | 10 ng/ml^d^ |
| C3-1876.8: YSIITPNILRLESEET | Light peptide | 1876.9 | - | - |
|  | Heavy peptide | 1883.8 | - | - |

a: The source-specific for LC-MS/MS parameters were as follows: ion spray voltage, 5500 V; curtain gas, 20 psi; nebulizer gas (GS1), 55 psi; auxiliary gas (GS2), 55 psi; source temperature, 550 ℃. b: DP, declustering potential, CE, collision energy; CXP, collision cell exit potential. c: internal standard (light peptide) concentraion used in clinical samples. d: internal standard (heavy peptide) concentraion used in calibrators.

**Supplementary Table 2** Demographic information and peptide candidates quantitation data for the discovery cohort

|  |  |  |  |  |  |  |  |
| --- | --- | --- | --- | --- | --- | --- | --- |
| sFlt-1 (pg/ml), PlGF (pg/ml) and peptide candidates quantitation (ng/ml) | | | | | | | |
| Internal ID | Group | sFlt-1 (pg/ml) | PlGF (pg/ml) | sFlt-1/PlGF | ITIH4-2026.9 | FGA-1033.4 | ITIH4-2051.1 |
| D-PE-1 | PE-positive | 4656 | 88.0 | 52.9 | 520.9 | 2.4 | 61.9 |
| D-PE-2 | PE-positive | 1394 | 409.4 | 3.4 | 401.6 | 5.9 | 45.2 |
| D-PE-3 | PE-positive | 3386 | 221.5 | 15.3 | 334.7 | 2.9 | 40.3 |
| D-PE-4 | PE-positive | 5189 | 556.0 | 9.3 | 551.8 | 5.6 | 74.9 |
| D-PE-5 | PE-positive | 794.4 | 202.1 | 3.9 | 465.1 | 3.5 | 46.8 |
| D-PE-6 | PE-positive | 2449 | 209.1 | 11.7 | 455.4 | 1.3 | 38.9 |
| D-PE-7 | PE-positive | 1185 | 303.7 | 3.9 | 358.9 | 6.2 | 42.6 |
| D-PE-8 | PE-positive | 2847 | 282.9 | 10.1 | 477.1 | 3.2 | 69.5 |
| D-PE-9 | PE-positive | 2168 | 271.8 | 8.0 | 382.0 | 1.5 | 42.1 |
| D-PE-10 | PE-positive | 26490 | 17.2 | 1540.1 | 434.0 | 5.2 | 53.3 |
| D-PE-11 | PE-positive | 1823 | 268.3 | 6.8 | 401.6 | 2.8 | 43.0 |
| D-PE-12 | PE-positive | 285.9 | 12.6 | 22.8 | 394.9 | 3.1 | 45.1 |
| D-PE-13 | PE-positive | 9298 | 221.1 | 42.1 | 431.0 | 2.5 | 44.9 |
| D-PE-14 | PE-positive | 4619 | 79.1 | 58.4 | 403.2 | 4.0 | 39.6 |
| D-PE-15 | PE-positive | 2178 | 30.0 | 72.6 | 419.5 | 2.0 | 45.1 |
| D-PE-16 | PE-positive | 3015 | 220.0 | 13.7 | 442.9 | 2.6 | 59.8 |
| D-PE-17 | PE-positive | 4794 | 69.1 | 69.4 | 516.2 | 7.6 | 80.9 |
| D-PE-18 | PE-positive | missing | missing | missing | 421.9 | 4.8 | 62.7 |
| D-PE-19 | PE-positive | 3176 | 540.8 | 5.9 | 473.9 | 4.4 | 41.6 |
| D-PE-20 | PE-positive | 7175 | 236.7 | 30.3 | 367.4 | 3.5 | 40.7 |
| D-PE-21 | PE-positive | 3629 | 137.4 | 26.4 | 417.7 | 1.3 | 35.6 |
| D-PE-22 | PE-positive | 2674 | 793.4 | 3.4 | 280.9 | 2.1 | 49.7 |
| D-PE-23 | PE-positive | 1340 | 653.1 | 2.1 | 288.2 | 6.6 | 28.0 |
| D-PE-24 | PE-positive | 1968 | 355.4 | 5.5 | 442.5 | 4.6 | 60.1 |
| D-PE-25 | PE-positive | 4178 | 738.8 | 5.7 | 400.6 | 5.8 | 44.4 |
| D-PE-26 | PE-positive | 1747 | 366.4 | 4.8 | 505.5 | 1.3 | 55.9 |
| D-PE-27 | PE-positive | 2221 | 272.4 | 8.2 | 401.9 | 2.1 | 52.6 |
| D-PE-28 | PE-positive | 2430 | 109.6 | 22.2 | 404.9 | 4.7 | 43.9 |
| D-PE-29 | PE-positive | 8595 | 44.8 | 191.9 | 446.4 | 1.3 | 44.3 |
| D-PE-30 | PE-positive | 4805 | 69.9 | 68.8 | 424.4 | 3.1 | 43.6 |
| D-CTR-1 | PE-negative | 1461 | 441.3 | 3.3 | 301.4 | 1.0 | 45.6 |
| D-CTR-2 | PE-negative | 201.3 | 80.5 | 2.5 | 205.97 | 0.67 | 17.84 |
| D-CTR-3 | PE-negative | 725.2 | 166.0 | 4.4 | 105.05 | 0.51 | 14.87 |
| D-CTR-4 | PE-negative | 3133 | 117.7 | 26.6 | 255.36 | 0.26 | 30.41 |
| D-CTR-5 | PE-negative | 2395 | 609.1 | 3.9 | 233.05 | 0.55 | 27.12 |
| D-CTR-6 | PE-negative | 3053 | 529.1 | 5.8 | 171.09 | 2.67 | 24.99 |
| D-CTR-7 | PE-negative | 1870 | 329.7 | 5.7 | 188.89 | 0.50 | 16.29 |
| D-CTR-8 | PE-negative | 1353 | 152.6 | 8.9 | 152.1 | 0.7 | 33.5 |
| D-CTR-9 | PE-negative | 1127 | 757.1 | 1.5 | 252.3 | 1.4 | 34.6 |
| D-CTR-10 | PE-negative | 1548 | 245.3 | 6.3 | 293.17 | 0.53 | 24.48 |
| D-CTR-11 | PE-negative | 3440 | 616.5 | 5.6 | 171.1 | 0.7 | 18.7 |
| D-CTR-12 | PE-negative | 3517 | 158.0 | 22.3 | 181.3 | 0.4 | 41.8 |
| D-CTR-13 | PE-negative | 3009 | 421.2 | 7.1 | 320.1 | 1.3 | 33.3 |
| D-CTR-14 | PE-negative | 3587 | 475.9 | 7.5 | 31.08 | 0.11 | 2.43 |
| D-CTR-15 | PE-negative | 1879 | 52.4 | 35.8 | 298.3 | 0.9 | 37.5 |
| D-CTR-16 | PE-negative | 2083 | 846.5 | 2.5 | 192.79 | 0.90 | 21.18 |
| D-CTR-17 | PE-negative | 4656 | 88.0 | 52.9 | 210.4 | 0.7 | 52.8 |
| D-CTR-18 | PE-negative | 1492 | 650.7 | 2.3 | 101.3 | 1.1 | 17.3 |
| D-CTR-19 | PE-negative | 6521 | 161.7 | 40.3 | 165.26 | 0.73 | 12.97 |
| D-CTR-20 | PE-negative | 2456 | 936.5 | 2.6 | 213.3 | 1.0 | 29.9 |
| D-CTR-21 | PE-negative | 1634 | 202.6 | 8.1 | 289.44 | 0.46 | 16.37 |
| D-CTR-22 | PE-negative | 2425 | 660.7 | 3.7 | 186.6 | 1.0 | 41.6 |
| D-CTR-23 | PE-negative | 1197 | 321.5 | 3.7 | 337.4 | 3.1 | 61.7 |
| D-CTR-24 | PE-negative | 2938 | 242.0 | 12.1 | 143.27 | 0.84 | 17.26 |
| D-CTR-25 | PE-negative | 2452 | 301.2 | 8.1 | 84.75 | 0.76 | 10.42 |
| D-CTR-26 | PE-negative | 1962 | 135.7 | 14.5 | 248.1 | 1.0 | 37.7 |
| D-CTR-27 | PE-negative | 3113 | 144.5 | 21.5 | 276.1 | 2.6 | 51.9 |
| D-CTR-28 | PE-negative | 11151 | 36.4 | 306.0 | 265.04 | 0.47 | 26.82 |
| D-CTR-29 | PE-negative | 2680 | 133.0 | 20.2 | 175.5 | 1.2 | 35.1 |
| D-CTR-30 | PE-negative | 997.5 | 1217.0 | 0.8 | 93.3 | 1.0 | 20.9 |
| D-CTR-31 | PE-negative | 1324 | 85.6 | 15.5 | 259.1 | 1.4 | 32.7 |
| D-CTR-32 | PE-negative | 2459 | 170.0 | 14.5 | 171.4 | 1.2 | 37.4 |
| D-CTR-33 | PE-negative | 5216 | 104.8 | 49.8 | 347.95 | 0.94 | 57.08 |
| D-CTR-34 | PE-negative | 1901 | 212.5 | 8.9 | 256.7 | 0.8 | 33.8 |
| D-CTR-35 | PE-negative | 1968 | 355.4 | 5.5 | 200.64 | 1.03 | 25.93 |
| D-CTR-36 | PE-negative | 1833 | 483.1 | 3.8 | 14.11 | 0.13 | 18.91 |
| D-CTR-37 | PE-negative | 860.9 | 366.1 | 2.4 | 158.5 | 1.1 | 34.6 |
| D-CTR-38 | PE-negative | 946.7 | 319.2 | 3.0 | 89.29 | 0.43 | 15.32 |
| D-CTR-39 | PE-negative | 3822 | 369.7 | 10.3 | 493.58 | 0.35 | 50.99 |
| D-CTR-40 | PE-negative | 2054 | 442.9 | 4.6 | 168.98 | 0.67 | 20.15 |
| D-CTR-41 | PE-negative | 3287 | 752.5 | 4.4 | 244.56 | 0.47 | 38.43 |
| D-CTR-42 | PE-negative | 3950 | 742.9 | 5.3 | 264.69 | 0.31 | 25.51 |
| D-CTR-43 | PE-negative | 7562 | 99.3 | 76.2 | 306.28 | 1.35 | 44.21 |
| D-CTR-44 | PE-negative | 1330 | 433.0 | 3.1 | 197.4 | 0.9 | 25.8 |
| D-CTR-45 | PE-negative | 2747 | 263.5 | 10.4 | 205.55 | 0.76 | 22.38 |
| D-CTR-46 | PE-negative | 1357 | 258.7 | 5.2 | 142.7 | 0.6 | 29.8 |
| D-CTR-47 | PE-negative | 6870 | 161.5 | 42.5 | 265.5 | 0.7 | 34.8 |
| D-CTR-48 | PE-negative | 1977 | 1271.0 | 1.6 | 181.85 | 0.83 | 13.85 |
| D-CTR-49 | PE-negative | 1952 | 889.0 | 2.2 | 207.47 | 0.29 | 18.86 |
| D-CTR-50 | PE-negative | 3482 | 96.0 | 36.3 | 319.5 | 2.3 | 40.7 |
| D-CTR-51 | PE-negative | 1591 | 801.0 | 2.0 | 272.6 | 1.4 | 32.8 |
| D-CTR-52 | PE-negative | 1981 | 531.1 | 3.7 | 139.55 | 0.88 | 19.54 |
| D-CTR-53 | PE-negative | 2487 | 227.4 | 10.9 | 169.0 | 1.6 | 41.0 |
| D-CTR-54 | PE-negative | 1949 | 595.9 | 3.3 | 232.0 | 1.4 | 34.9 |
| D-CTR-55 | PE-negative | 1675 | 962.2 | 1.7 | 347.0 | 2.4 | 50.4 |
| D-CTR-56 | PE-negative | 2000 | 148.9 | 13.4 | 225.28 | 0.79 | 17.51 |
| D-CTR-57 | PE-negative | 736.1 | 324.0 | 2.3 | 65.3 | 0.7 | 13.4 |
| D-CTR-58 | PE-negative | 2756 | 582.7 | 4.7 | 403.23 | 0.63 | 43.07 |
| D-CTR-59 | PE-negative | 2676 | 113.2 | 23.6 | 56.59 | 2.06 | 6.11 |
| D-CTR-60 | PE-negative | 4130 | 585.4 | 7.1 | 120.38 | 0.18 | 6.51 |
| D-CTR-61 | PE-negative | 1217 | 758.8 | 1.6 | 212.49 | 0.86 | 11.60 |
| D-CTR-62 | PE-negative | 1546 | 293.9 | 5.3 | 341.5 | 2.2 | 57.4 |
| D-CTR-63 | PE-negative | 382.8 | 73.3 | 5.2 | 215.5 | 2.8 | 37.7 |
| D-CTR-64 | PE-negative | 741.1 | 207.1 | 3.6 | 317.66 | 1.19 | 29.28 |
| D-CTR-65 | PE-negative | 2189 | 45.7 | 47.9 | 256.3 | 2.0 | 51.0 |
| D-CTR-66 | PE-negative | 1954 | 48.7 | 40.1 | 213.17 | 1.79 | 34.13 |
| D-CTR-67 | PE-negative | 2274 | 443.8 | 5.1 | 649.35 | 0.93 | 72.20 |
| D-CTR-68 | PE-negative | 2043 | 344.3 | 5.9 | 177.7 | 2.0 | 43.4 |
| D-CTR-69 | PE-negative | 1179 | 12.8 | 92.0 | 301.20 | 0.71 | 28.76 |
| D-CTR-70 | PE-negative | 1018 | 347.0 | 2.9 | 84.46 | 0.42 | 12.94 |
| D-CTR-71 | PE-negative | 896.3 | 13.9 | 64.4 | 169.1 | 1.5 | 45.7 |
| D-CTR-72 | PE-negative | 2864 | 130.6 | 21.9 | 286.4 | 1.2 | 51.2 |
| D-CTR-73 | PE-negative | 2232 | 657.7 | 3.4 | 257.07 | 1.09 | 28.36 |
| D-CTR-74 | PE-negative | 2344 | 23.5 | 99.6 | 104.13 | 0.75 | 12.67 |
| D-CTR-75 | PE-negative | 4705 | 50.5 | 93.1 | 81.17 | 0.75 | 10.09 |
| D-CTR-76 | PE-negative | 1866 | 96.0 | 19.4 | 398.7 | 3.1 | 60.8 |
| D-CTR-77 | PE-negative | 3090 | 102.9 | 30.0 | 156.3 | 0.9 | 45.6 |
| D-CTR-78 | PE-negative | 2036 | 250.9 | 8.1 | 181.36 | 0.61 | 26.70 |
| D-CTR-79 | PE-negative | 2543 | 375.4 | 6.8 | 241.5 | 1.1 | 47.5 |
| D-CTR-80 | PE-negative | 1194 | 687.8 | 1.7 | 234.7 | 1.3 | 53.9 |
| D-CTR-81 | PE-negative | 1880 | 396.3 | 4.7 | 386.1 | 3.0 | 73.4 |
| D-CTR-82 | PE-negative | 1128 | 82.2 | 13.7 | 175.28 | 0.55 | 30.72 |
| D-CTR-83 | PE-negative | 9767 | 160.6 | 60.8 | 293.9 | 1.3 | 33.7 |
| D-CTR-84 | PE-negative | 5410 | 301.3 | 18.0 | 199.56 | 0.23 | 38.85 |
| D-CTR-85 | PE-negative | 1559 | 313.2 | 5.0 | 460.83 | 0.50 | 39.14 |
|  |  |  |  |  |  |  |  |

**Supplementary Table 2** Demographic information and peptide candidates quantitation data for the discovery cohort (continued)

|  |  |  |  |  |  |  |
| --- | --- | --- | --- | --- | --- | --- |
| Demographic information for the discovery cohort | | | | | | |
| Internal ID | Group | Age | pre-pregnancy BMI | Gravidity | Parity | Sampling week |
| D-PE-1 | PE-positive | 39 | 24.5 | 2 | 1 | 25 |
| D-PE-2 | PE-positive | 37 | 28.2 | 3 | 0 | 35 |
| D-PE-3 | PE-positive | 30 | 25.7 | 1 | 0 | 31 |
| D-PE-4 | PE-positive | 35 | 28.7 | 2 | 0 | 29 |
| D-PE-5 | PE-positive | 31 | 23.2 | 4 | 2 | 21 |
| D-PE-6 | PE-positive | 31 | 34.5 | 1 | 0 | 30 |
| D-PE-7 | PE-positive | 38 | 23.0 | 2 | 1 | 32 |
| D-PE-8 | PE-positive | 37 | 28.2 | 3 | 1 | 23 |
| D-PE-9 | PE-positive | 40 | 20.9 | 3 | 1 | 28 |
| D-PE-10 | PE-positive | 27 | 28.3 | 3 | 0 | 32 |
| D-PE-11 | PE-positive | 37 | 24.5 | 1 | 0 | 23 |
| D-PE-12 | PE-positive | 27 | 20.1 | 1 | 0 | 26 |
| D-PE-13 | PE-positive | 31 | 30.8 | 1 | 0 | 23 |
| D-PE-14 | PE-positive | 29 | 19.5 | 2 | 0 | 32 |
| D-PE-15 | PE-positive | 29 | 23.7 | 1 | 0 | 25 |
| D-PE-16 | PE-positive | 27 | missing | 1 | 0 | 24 |
| D-PE-17 | PE-positive | 36 | 27.0 | 2 | 1 | 34 |
| D-PE-18 | PE-positive | 33 | 20.9 | 3 | 0 | 36 |
| D-PE-19 | PE-positive | 30 | 29.4 | 1 | 0 | 25 |
| D-PE-20 | PE-positive | 36 | 23.0 | 3 | 0 | 25 |
| D-PE-21 | PE-positive | 35 | 31.1 | 2 | 1 | 29 |
| D-PE-22 | PE-positive | 32 | 22.7 | 2 | 0 | 23 |
| D-PE-23 | PE-positive | 30 | 22.8 | 3 | 0 | 26 |
| D-PE-24 | PE-positive | 39 | 24.8 | 3 | 0 | 33 |
| D-PE-25 | PE-positive | 30 | 22.6 | 2 | 1 | 25 |
| D-PE-26 | PE-positive | 32 | 23.9 | 2 | 0 | 26 |
| D-PE-27 | PE-positive | 33 | missing | 1 | 0 | 30 |
| D-PE-28 | PE-positive | 40 | 22.0 | 2 | 1 | 36 |
| D-PE-29 | PE-positive | 35 | 37.0 | 4 | 1 | 32 |
| D-PE-30 | PE-positive | 35 | 19.5 | 1 | 0 | 32 |
| D-CTR-1 | PE-negative | 28 | 24.4 | 6 | 1 | 25 |
| D-CTR-2 | PE-negative | 30 | missing | 2 | 0 | 23 |
| D-CTR-3 | PE-negative | 43 | missing | 4 | 1 | 35 |
| D-CTR-4 | PE-negative | 42 | 24.6 | 3 | 1 | 36 |
| D-CTR-5 | PE-negative | 30 | 22.4 | 1 | 0 | 34 |
| D-CTR-6 | PE-negative | 32 | 28.7 | 4 | 0 | 25 |
| D-CTR-7 | PE-negative | 37 | 22.3 | 1 | 0 | 34 |
| D-CTR-8 | PE-negative | 28 | 21.5 | 2 | 0 | 20 |
| D-CTR-9 | PE-negative | 32 | 27.6 | 1 | 0 | 26 |
| D-CTR-10 | PE-negative | 35 | 22.3 | 1 | 0 | 22 |
| D-CTR-11 | PE-negative | 29 | 19.1 | 1 | 0 | 26 |
| D-CTR-12 | PE-negative | 33 | missing | 1 | 0 | 25 |
| D-CTR-13 | PE-negative | 28 | 16.0 | 1 | 0 | 25 |
| D-CTR-14 | PE-negative | 29 | 21.0 | 1 | 0 | 32 |
| D-CTR-15 | PE-negative | 31 | 34.5 | 1 | 0 | 30 |
| D-CTR-16 | PE-negative | 36 | 22.6 | 2 | 0 | 27 |
| D-CTR-17 | PE-negative | 36 | 19.7 | 1 | 0 | 28 |
| D-CTR-18 | PE-negative | 33 | 20.9 | 3 | 1 | 33 |
| D-CTR-19 | PE-negative | 34 | 23.8 | 2 | 0 | 23 |
| D-CTR-20 | PE-negative | 34 | 25.0 | 2 | 0 | 34 |
| D-CTR-21 | PE-negative | 33 | missing | 1 | 0 | 34 |
| D-CTR-22 | PE-negative | 37 | 40.5 | 6 | 1 | 27 |
| D-CTR-23 | PE-negative | 27 | 20.2 | 2 | 1 | 33 |
| D-CTR-24 | PE-negative | 38 | 20.9 | 1 | 0 | 34 |
| D-CTR-25 | PE-negative | 39 | 26.2 | 6 | 1 | 27 |
| D-CTR-26 | PE-negative | 35 | 24.6 | 1 | 0 | 33 |
| D-CTR-27 | PE-negative | 32 | 32.1 | 3 | 0 | 34 |
| D-CTR-28 | PE-negative | 34 | 26.8 | 2 | 1 | 28 |
| D-CTR-29 | PE-negative | 27 | 21.8 | 1 | 0 | 26 |
| D-CTR-30 | PE-negative | 29 | 32.3 | 1 | 0 | 27 |
| D-CTR-31 | PE-negative | 32 | 26.0 | 2 | 0 | 35 |
| D-CTR-32 | PE-negative | 27 | 26.5 | 1 | 0 | 36 |
| D-CTR-33 | PE-negative | 43 | 30.4 | 5 | 1 | 35 |
| D-CTR-34 | PE-negative | 44 | 26.9 | 2 | 1 | 33 |
| D-CTR-35 | PE-negative | 30 | 23.2 | 1 | 0 | 24 |
| D-CTR-36 | PE-negative | 37 | 24.0 | 1 | 0 | 36 |
| D-CTR-37 | PE-negative | 34 | 22.1 | 3 | 1 | 22 |
| D-CTR-38 | PE-negative | 28 | 18.1 | 1 | 0 | 35 |
| D-CTR-39 | PE-negative | missing | missing | missing | missing | 34 |
| D-CTR-40 | PE-negative | 37 | 25.4 | 4 | 1 | 22 |
| D-CTR-41 | PE-negative | 34 | 29.8 | 2 | 1 | 21 |
| D-CTR-42 | PE-negative | 35 | 20.2 | 2 | 0 | 31 |
| D-CTR-43 | PE-negative | 35 | 32.4 | 2 | 1 | 28 |
| D-CTR-44 | PE-negative | 33 | 30.5 | 3 | 1 | 31 |
| D-CTR-45 | PE-negative | missing | missing | missing | missing | 35 |
| D-CTR-46 | PE-negative | 29 | 23.7 | 1 | 0 | 36 |
| D-CTR-47 | PE-negative | 29 | 23.9 | 2 | 0 | 36 |
| D-CTR-48 | PE-negative | 41 | 24.8 | 4 | 1 | 27 |
| D-CTR-49 | PE-negative | 34 | 24.6 | 1 | 0 | 30 |
| D-CTR-50 | PE-negative | 30 | 20.6 | 1 | 0 | 31 |
| D-CTR-51 | PE-negative | 36 | 21.2 | 4 | 0 | 28 |
| D-CTR-52 | PE-negative | 35 | 22.4 | 2 | 1 | 35 |
| D-CTR-53 | PE-negative | 29 | missing | 1 | 0 | 25 |
| D-CTR-54 | PE-negative | 35 | 19.1 | 2 | 0 | 34 |
| D-CTR-55 | PE-negative | 35 | missing | 2 | 1 | 37 |
| D-CTR-56 | PE-negative | 32 | 19.9 | 3 | 1 | 24 |
| D-CTR-57 | PE-negative | 29 | 21.7 | 1 | 0 | 32 |
| D-CTR-58 | PE-negative | 39 | missing | 5 | 2 | 36 |
| D-CTR-59 | PE-negative | 36 | 24.6 | 2 | 1 | 21 |
| D-CTR-60 | PE-negative | 29 | 28.0 | 1 | 0 | 31 |
| D-CTR-61 | PE-negative | 36 | 23.4 | 3 | 0 | 33 |
| D-CTR-62 | PE-negative | 26 | 22.0 | 2 | 0 | 20 |
| D-CTR-63 | PE-negative | 28 | 21.5 | 1 | 0 | 33 |
| D-CTR-64 | PE-negative | 35 | 29.1 | 2 | 0 | 27 |
| D-CTR-65 | PE-negative | 35 | 29.2 | 2 | 0 | 21 |
| D-CTR-66 | PE-negative | missing | missing | missing | missing | 24 |
| D-CTR-67 | PE-negative | 29 | 20.0 | 1 | 0 | 28 |
| D-CTR-68 | PE-negative | 31 | 19.2 | 2 | 0 | 23 |
| D-CTR-69 | PE-negative | 30 | 22.0 |  |  | 31 |
| D-CTR-70 | PE-negative | 26 | 25.2 | 2 | 0 | 34 |
| D-CTR-71 | PE-negative | 30 | 24.0 | 1 | 0 | 23 |
| D-CTR-72 | PE-negative | 26 | 24.0 | 1 | 0 | 22 |
| D-CTR-73 | PE-negative | 38 | 21.5 | 2 | 0 | 20 |
| D-CTR-74 | PE-negative | 26 | 24.5 | 1 | 0 | 34 |
| D-CTR-75 | PE-negative | 32 | 22.7 | 1 | 0 | 21 |
| D-CTR-76 | PE-negative | 37 | 21.1 | 3 | 1 | 22 |
| D-CTR-77 | PE-negative | 30 | missing | missing | missing | 27 |
| D-CTR-78 | PE-negative | 33 | 22.3 | 1 | 0 | 23 |
| D-CTR-79 | PE-negative | 34 | missing | missing | missing | 33 |
| D-CTR-80 | PE-negative | 31 | 21.6 | 1 | 0 | 21 |
| D-CTR-81 | PE-negative | 30 | 18.9 | 3 | 1 | 34 |
| D-CTR-82 | PE-negative | 30 | 20.6 | 1 | 0 | 32 |
| D-CTR-83 | PE-negative | 43 | 26.0 | 4 | 1 | 20 |
| D-CTR-84 | PE-negative | 36 | 24.6 | 4 | 2 | 32 |
| D-CTR-85 | PE-negative | 31 | 30.9 | 2 | 0 | 35 |
|  |  |  |  |  |  |  |
|  |  |  |  |  |  |  |

**Supplementary Table 3** Demographic information and peptide candidates quantitation data for the validation cohort

|  |  |  |  |  |  |  |  |
| --- | --- | --- | --- | --- | --- | --- | --- |
| sFlt-1 (pg/ml), PlGF (pg/ml) and peptide candidates quantitation (ng/ml) | | | | | | | |
| Internal ID | Group | sFlt-1 (pg/ml) | PlGF (pg/ml) | sFlt-1/PlGF | ITIH4-2026.9 | FGA-1033.4 | ITIH4-2051.1 |
| V-PE-1 | PE-positive | 2168 | 271.8 | 8.0 | 483.1 | 3.1 | 70.6 |
| V-PE-2 | PE-positive | 5358 | 92.8 | 57.7 | 291.2 | 0.9 | 18.4 |
| V-PE-3 | PE-positive | 2678 | 128.8 | 20.8 | 268.1 | 2.9 | 20.2 |
| V-PE-4 | PE-positive | 2814 | 766.2 | 3.7 | 443.7 | 3.0 | 58.9 |
| V-PE-5 | PE-positive | 4553 | 487.9 | 9.3 | 520.3 | 3.7 | 73.9 |
| V-PE-6 | PE-positive | 1677 | 245.3 | 6.8 | 174.0 | 0.2 | 17.9 |
| V-PE-7 | PE-positive | 7855 | 10.2 | 769.3 | 407.8 | 6.1 | 50.4 |
| V-PE-8 | PE-positive | 2034 | 226.5 | 9.0 | 468.2 | 2.5 | 65.1 |
| V-PE-9 | PE-positive | 3742 | 32.5 | 115.3 | 388.2 | 3.8 | 53.0 |
| V-PE-10 | PE-positive | 2162 | 28.7 | 75.3 | 418.8 | 1.8 | 26.7 |
| V-PE-11 | PE-positive | 1447 | 124.9 | 11.6 | 500.9 | 5.3 | 69.7 |
| V-PE-12 | PE-positive | 3240 | 243.2 | 13.3 | 414.3 | 3.9 | 62.4 |
| V-PE-13 | PE-positive | 2436 | 209.3 | 11.6 | 380.8 | 4.6 | 39.0 |
| V-PE-14 | PE-positive | 1925 | 320.0 | 6.0 | 291.7 | 1.2 | 24.3 |
| V-PE-15 | PE-positive | 1219 | 111.7 | 10.9 | 280.1 | 1.8 | 34.0 |
| V-PE-16 | PE-positive | 13085 | 30.2 | 434.0 | 375.4 | 2.0 | 48.8 |
| V-PE-17 | PE-positive | 6067 | 38.7 | 156.6 | 419.1 | 2.7 | 43.1 |
| V-PE-18 | PE-positive | 585.2 | 9.6 | 61.2 | 362.1 | 1.4 | 38.3 |
| V-PE-19 | PE-positive | 3929 | 167.7 | 23.4 | 360.5 | 2.0 | 37.0 |
| V-PE-20 | PE-positive | 22785 | 5.3 | 4315.3 | 439.4 | 3.7 | 48.1 |
| V-CTR-1 | PE-negative | 480.1 | 271.5 | 1.8 | 118.1 | 0.8 | 28.4 |
| V-CTR-2 | PE-negative | 1676 | 394.3 | 4.3 | 298.42 | 0.65 | 21.44 |
| V-CTR-3 | PE-negative | 19046 | 96.3 | 197.8 | 210.35 | 1.27 | 21.03 |
| V-CTR-4 | PE-negative | 2097 | 472.1 | 4.4 | 92.25 | 0.46 | 9.71 |
| V-CTR-5 | PE-negative | 2029 | 172.8 | 11.7 | 243.3 | 1.3 | 43.3 |
| V-CTR-6 | PE-negative | 12343 | 338.3 | 36.5 | 257.33 | 0.49 | 25.85 |
| V-CTR-7 | PE-negative | 2157 | 137.3 | 15.7 | 115.46 | 0.42 | 6.96 |
| V-CTR-8 | PE-negative | 1549 | 511.9 | 3.0 | 221.14 | 1.54 | 18.12 |
| V-CTR-9 | PE-negative | 1476 | 424.1 | 3.5 | 141.7 | 0.9 | 37.6 |
| V-CTR-10 | PE-negative | 3267 | 351.4 | 9.3 | 308.8 | 1.5 | 48.7 |
| V-CTR-11 | PE-negative | 1319 | 663.2 | 2.0 | 361.27 | 0.88 | 41.20 |
| V-CTR-12 | PE-negative | 7296 | 200.1 | 36.5 | 142.21 | 0.51 | 26.65 |
| V-CTR-13 | PE-negative | 3275 | 460.4 | 7.1 | 94.34 | 0.39 | 6.54 |
| V-CTR-14 | PE-negative | 1366 | 515.4 | 2.7 | 110.22 | 0.60 | 15.84 |
| V-CTR-15 | PE-negative | 4205 | 350.0 | 12.0 | 138.22 | 0.67 | 13.29 |
| V-CTR-16 | PE-negative | 3777 | 100.9 | 37.4 | 222.67 | 0.70 | 21.70 |
| V-CTR-17 | PE-negative | 6250 | 262.7 | 23.8 | 321.75 | 0.40 | 31.81 |
| V-CTR-18 | PE-negative | 1779 | 1553.0 | 1.1 | 298.69 | 0.83 | 24.92 |
| V-CTR-19 | PE-negative | 5338 | 164.7 | 32.4 | 267.17 | 0.93 | 47.13 |
| V-CTR-20 | PE-negative | 1481 | 429.9 | 3.4 | 469.0 | 4.2 | 65.2 |
| V-CTR-21 | PE-negative | 1853 | 578.1 | 3.2 | 448.63 | 2.13 | 44.23 |
| V-CTR-22 | PE-negative | 2595 | 109.6 | 23.7 | 427.0 | 1.1 | 48.7 |
| V-CTR-23 | PE-negative | 1992 | 290.7 | 6.9 | 133.4 | 0.9 | 30.5 |
| V-CTR-24 | PE-negative | 1348 | 301.4 | 4.5 | 267.24 | 1.21 | 36.83 |
| V-CTR-25 | PE-negative | 1720 | 147.2 | 11.7 | 343.29 | 1.53 | 37.12 |
| V-CTR-26 | PE-negative | 3540 | 494.9 | 7.2 | 252.40 | 1.58 | 33.22 |
| V-CTR-27 | PE-negative | 969.5 | 247.3 | 3.9 | 10.91 | 0.09 | 1.51 |
| V-CTR-28 | PE-negative | 1792 | 184.3 | 9.7 | 214.5 | 1.0 | 50.5 |
| V-CTR-29 | PE-negative | 1069 | 113.8 | 9.4 | 276.40 | 1.00 | 32.38 |
| V-CTR-30 | PE-negative | 1726 | 359.2 | 4.8 | 293.69 | 3.51 | 28.31 |
| V-CTR-31 | PE-negative | 1757 | 715.9 | 2.5 | 292.40 | 0.71 | 41.72 |
| V-CTR-32 | PE-negative | 3374 | 992.1 | 3.4 | 247.28 | 0.30 | 19.64 |
| V-CTR-33 | PE-negative | 2114 | 560.6 | 3.8 | 147.69 | 0.62 | 5.05 |
| V-CTR-34 | PE-negative | 1323 | 566.9 | 2.3 | 179.92 | 0.87 | 26.68 |
| V-CTR-35 | PE-negative | 475.6 | 550.7 | 0.9 | 323.4 | 1.8 | 37.6 |
| V-CTR-36 | PE-negative | 1633 | 377.7 | 4.3 | 289.35 | 1.17 | 48.97 |
| V-CTR-37 | PE-negative | 2142 | 584.0 | 3.7 | 293.3 | 0.9 | 33.0 |
| V-CTR-38 | PE-negative | 1661 | 543.7 | 3.1 | 216.17 | 0.77 | 31.82 |
| V-CTR-39 | PE-negative | 800.4 | 21.5 | 37.2 | 203.25 | 0.52 | 21.32 |
| V-CTR-40 | PE-negative | 1698 | 1190.0 | 1.4 | 239.3 | 0.4 | 40.8 |
| V-CTR-41 | PE-negative | 2209 | 67.5 | 32.7 | 188.36 | 0.90 | 13.81 |
| V-CTR-42 | PE-negative | 1804 | 338.8 | 5.3 | 284.6 | 0.6 | 31.9 |
| V-CTR-43 | PE-negative | 367.4 | 186.5 | 2.0 | 264.62 | 0.91 | 50.05 |
| V-CTR-44 | PE-negative | 2585 | 483.3 | 5.3 | 340.25 | 0.46 | 36.06 |
| V-CTR-45 | PE-negative | 1940 | 17.2 | 113.1 | 221.09 | 0.81 | 12.02 |
| V-CTR-46 | PE-negative | 2310 | 251.3 | 9.2 | 334.78 | 1.28 | 40.14 |
| V-CTR-47 | PE-negative | 1648 | 339.7 | 4.9 | 203.33 | 0.79 | 22.76 |
| V-CTR-48 | PE-negative | 3849 | 29.5 | 130.4 | 268.24 | 0.56 | 36.44 |
| V-CTR-49 | PE-negative | 2196 | 454.0 | 4.8 | 329.71 | 1.00 | 38.39 |
| V-CTR-50 | PE-negative | 4355 | 428.3 | 10.2 | 185.98 | 0.42 | 22.69 |
| V-CTR-51 | PE-negative | 2797 | 849.7 | 3.3 | 221.68 | 0.75 | 39.57 |
| V-CTR-52 | PE-negative | 2157 | 99.6 | 21.7 | 188.93 | 0.52 | 18.42 |
| V-CTR-53 | PE-negative | 6582 | 98.4 | 66.9 | 55.10 | 0.80 | 15.44 |
| V-CTR-54 | PE-negative | 1986 | 139.2 | 14.3 | 134.08 | 0.80 | 18.26 |
| V-CTR-55 | PE-negative | 3240 | 591.6 | 5.5 | 175.84 | 0.36 | 23.82 |
| V-CTR-56 | PE-negative | 6423 | 20.2 | 318.6 | 375.23 | 0.73 | 42.75 |
| V-CTR-57 | PE-negative | 3022 | 169.9 | 17.8 | 202.06 | 1.05 | 28.65 |
| V-CTR-58 | PE-negative | 1619 | 56.1 | 28.9 | 281.45 | 1.17 | 39.68 |
| V-CTR-59 | PE-negative | 2906 | 463.4 | 6.3 | 338.07 | 0.69 | 47.26 |
| V-CTR-60 | PE-negative | 4569 | 69.7 | 65.5 | 100.26 | 0.70 | 16.64 |
|  |  |  |  |  |  |  |  |

**Supplementary Table 3** Demographic information and peptide candidates quantitation data for the validation cohort (continued)

|  |  |  |  |  |  |  |
| --- | --- | --- | --- | --- | --- | --- |
| Demographic information for the validation cohort | | | | | | |
| Internal ID | Group | Age | Pre-pregnancy BMI | Gravidity | Parity | Sampling week |
| V-PE-1 | PE-positive | 40 | 20.9 | 3 | 1 | 28 |
| V-PE-2 | PE-positive | 28 | missing | 1 | 0 | 23 |
| V-PE-3 | PE-positive | 42 | 23.4 | 5 | 1 | 27 |
| V-PE-4 | PE-positive | 41 | 24.7 | 4 | 1 | 28 |
| V-PE-5 | PE-positive | 30 | 27.7 | 1 | 0 | 31 |
| V-PE-6 | PE-positive | 29 | missing | 3 | 1 | 30 |
| V-PE-7 | PE-positive | 30 | 26.1 | 3 | 0 | 31 |
| V-PE-8 | PE-positive | 38 | 21.3 | 2 | 0 | 29 |
| V-PE-9 | PE-positive | 26 | 18.5 | 1 | 0 | 31 |
| V-PE-10 | PE-positive | 29 | missing | 1 | 0 | 32 |
| V-PE-11 | PE-positive | 40 | 24.2 | 5 | 1 | 34 |
| V-PE-12 | PE-positive | 35 | 32.4 | 2 | 1 | 22 |
| V-PE-13 | PE-positive | 31 | 20.6 | 4 | 0 | 31 |
| V-PE-14 | PE-positive | 25 | 29.8 | 1 | 0 | 32 |
| V-PE-15 | PE-positive | 36 | 27.6 | 2 | 1 | 31 |
| V-PE-16 | PE-positive | 34 | 23.2 | 1 | 0 | 33 |
| V-PE-17 | PE-positive | 30 | 17.7 | 2 | 0 | 32 |
| V-PE-18 | PE-positive | 34 | 26.4 | 1 | 0 | 31 |
| V-PE-19 | PE-positive | 31 | 30.8 | 1 | 0 | 30 |
| V-PE-20 | PE-positive | 22 | 21.3 | 2 | 0 | 26 |
| V-CTR-1 | PE-negative | 28 | 22.5 | 3 | 1 | 21 |
| V-CTR-2 | PE-negative | 38 | 24.4 | 3 | 1 | 34 |
| V-CTR-3 | PE-negative | 30 | 17.8 | 1 | 0 | 23 |
| V-CTR-4 | PE-negative | 31 | 30.0 | 2 | 0 | 26 |
| V-CTR-5 | PE-negative | 34 | 24.8 | 2 | 1 | 36 |
| V-CTR-6 | PE-negative | 39 | 24.5 | 2 | 1 | 31 |
| V-CTR-7 | PE-negative | 30 | 20.1 | 2 | 0 | 28 |
| V-CTR-8 | PE-negative | 34 | 30.6 | 1 | 0 | 35 |
| V-CTR-9 | PE-negative | 33 | 22.3 | 1 | 0 | 31 |
| V-CTR-10 | PE-negative | 39 | 27.1 | 6 | 2 | 34 |
| V-CTR-11 | PE-negative | 36 | 24.5 | 2 | 1 | 20 |
| V-CTR-12 | PE-negative | 37 | 40.5 | 6 | 1 | 25 |
| V-CTR-13 | PE-negative | 29 | missing | 1 | 0 | 25 |
| V-CTR-14 | PE-negative | 29 | missing | 2 | 0 | 30 |
| V-CTR-15 | PE-negative | 34 | 30.1 | 2 | 0 | 24 |
| V-CTR-16 | PE-negative | 35 | 29.1 | 2 | 0 | 34 |
| V-CTR-17 | PE-negative | 35 | 19.5 | 3 | 1 | 29 |
| V-CTR-18 | PE-negative | missing | missing | missing | missing | 32 |
| V-CTR-19 | PE-negative | 30 | 22.3 | 1 | 0 | 24 |
| V-CTR-20 | PE-negative | 33 | 20.9 | 2 | 1 | 31 |
| V-CTR-21 | PE-negative | 35 | 24.8 | 2 | 0 | 35 |
| V-CTR-22 | PE-negative | 37 | 22.4 | 2 | 1 | 30 |
| V-CTR-23 | PE-negative | 39 | 28.1 | 4 | 0 | 20 |
| V-CTR-24 | PE-negative | 44 | 26.5 | 2 | 1 | 27 |
| V-CTR-25 | PE-negative | 34 | 30.1 | 1 | 0 | 27 |
| V-CTR-26 | PE-negative | 30 | 21.3 | 2 | 0 | 31 |
| V-CTR-27 | PE-negative | 36 | missing | 2 | 1 | 33 |
| V-CTR-28 | PE-negative | 43 | missing | missing | missing | 21 |
| V-CTR-29 | PE-negative | 32 | 38.0 | 3 | 0 | 29 |
| V-CTR-30 | PE-negative | 29 | 24.8 | 1 | 0 | 29 |
| V-CTR-31 | PE-negative | missing | missing | missing | missing | 26 |
| V-CTR-32 | PE-negative | 34 | 18.6 | 1 | 0 | 24 |
| V-CTR-33 | PE-negative | 33 | 23.4 | 2 | 0 | 33 |
| V-CTR-34 | PE-negative | 37 | 23.0 | 3 | 1 | 34 |
| V-CTR-35 | PE-negative | 31 | 21.5 | 1 | 0 | 23 |
| V-CTR-36 | PE-negative | 37 | 25.4 | 6 | 1 | 31 |
| V-CTR-37 | PE-negative | 41 | 21.4 | 4 | 1 | 21 |
| V-CTR-38 | PE-negative | 29 | 24.6 | 6 | 1 | 25 |
| V-CTR-39 | PE-negative | 35 | 26.0 | 3 | 0 | 23 |
| V-CTR-40 | PE-negative | 31 | 23.5 | 2 | 0 | 27 |
| V-CTR-41 | PE-negative | 39 | 29.3 | 1 | 0 | 34 |
| V-CTR-42 | PE-negative | 30 | 27.3 | 1 | 0 | 20 |
| V-CTR-43 | PE-negative | 32 | 18.9 | 1 | 0 | 31 |
| V-CTR-44 | PE-negative | 26 | 24.2 | 1 | 0 | 25 |
| V-CTR-45 | PE-negative | 30 | 21.0 | 1 | 0 | 23 |
| V-CTR-46 | PE-negative | missing | missing | missing | missing | 25 |
| V-CTR-47 | PE-negative | 26 | 21.3 | 2 | 1 | 25 |
| V-CTR-48 | PE-negative | 42 | 24.6 | 3 | 1 | 22 |
| V-CTR-49 | PE-negative | 34 | missing | 1 | 0 | 29 |
| V-CTR-50 | PE-negative | 30 | 29.4 | 1 | 0 | 34 |
| V-CTR-51 | PE-negative | missing | missing | missing | missing | 28 |
| V-CTR-52 | PE-negative | missing | missing | missing | missing | 32 |
| V-CTR-53 | PE-negative | 39 | 21.2 | 1 | 0 | 33 |
| V-CTR-54 | PE-negative | 29 | 28.0 | 1 | 0 | 28 |
| V-CTR-55 | PE-negative | missing | missing | missing | missing | 35 |
| V-CTR-56 | PE-negative | 29 | 29.1 | 2 | 0 | 29 |
| V-CTR-57 | PE-negative | 39 | 22.3 | 4 | 0 | 24 |
| V-CTR-58 | PE-negative | 32 | 32.8 | 2 | 1 | 35 |
| V-CTR-59 | PE-negative | 38 | 20.9 | 3 | 1 | 32 |
| V-CTR-60 | PE-negative | 30 | 27.3 | 1 | 0 | 29 |
|  |  |  |  |  |  |  |

**Supplementary Table 4** Significantly differentially expressed mass peaks that were unidentifiable in LTQ-Orbitrap-MS

|  |  |  |  |  |  |  |
| --- | --- | --- | --- | --- | --- | --- |
|  |  | PE-positive (n=30) | | PE-negasitive (n=30) | |  |
| Mass peak, *m/z*^a^ | FDR-adjusted *p* value | Mean | SD^b^ | Mean | SD | FC^c^ |
| 2012.8 | 1.02E-09 | 2210.5 | 1283.3 | 39.3 | 46.9 | 56.25 |
| 2004.4 | 1.02E-09 | 2173.0 | 1691.8 | 58.6 | 52.2 | 37.08 |
| 1847.6 | 1.02E-09 | 370.8 | 267.2 | 13.9 | 12.6 | 26.67 |
| 1987.3 | 1.43E-09 | 114.0 | 51.5 | 22.7 | 18.4 | 5.03 |
| 4287.2 | 1.02E-09 | 1042.2 | 315.6 | 277.5 | 182.9 | 3.76 |
| 4295.4 | 3.60E-09 | 786.5 | 151.9 | 263.0 | 185.3 | 2.99 |
| 4064.1 | 3.56E-06 | 305.4 | 195.7 | 103.8 | 84.4 | 2.94 |
| 2109.5 | 3.17E-07 | 148.2 | 96.1 | 52.2 | 51.6 | 2.84 |
| 4055.0 | 3.94E-05 | 308.3 | 213.2 | 115.0 | 88.4 | 2.68 |
| 8150.7 | 3.94E-05 | 246.6 | 161.3 | 92.4 | 82.4 | 2.67 |
| 2064.8 | 4.70E-05 | 502.1 | 361.2 | 197.4 | 169.0 | 2.54 |
| 3672.9 | 5.89E-08 | 152.7 | 64.4 | 60.4 | 27.1 | 2.53 |
| 3957.0 | 2.50E-03 | 818.5 | 656.0 | 325.6 | 209.3 | 2.51 |
| 8140.9 | 5.16E-04 | 254.3 | 176.8 | 108.2 | 108.1 | 2.35 |
| 1948.8 | 8.20E-07 | 188.0 | 71.0 | 82.0 | 47.7 | 2.29 |
| 4036.2 | 3.02E-05 | 103.0 | 55.7 | 45.4 | 38.7 | 2.27 |
| 2099.1 | 8.11E-07 | 209.9 | 103.6 | 93.1 | 60.9 | 2.25 |
| 1960.8 | 4.77E-07 | 125.7 | 53.9 | 55.8 | 24.9 | 2.25 |
| 4257.0 | 8.11E-07 | 354.4 | 167.4 | 162.9 | 137.3 | 2.18 |
| 3513.8 | 4.41E-08 | 125.7 | 40.4 | 58.5 | 28.1 | 2.15 |
| 4086.0 | 3.56E-06 | 327.1 | 134.4 | 152.3 | 103.5 | 2.15 |
| 4077.7 | 3.51E-06 | 317.7 | 128.8 | 148.2 | 95.7 | 2.14 |
| 3806.6 | 1.41E-09 | 142.5 | 42.4 | 67.1 | 29.9 | 2.12 |
| 8132.4 | 9.90E-03 | 207.0 | 172.3 | 102.3 | 101.3 | 2.02 |
| a: protonated mass/charge ratio by MALDI-TOF; b: standard deviation; c: fold change. | | | | |  |  |

**Supplementary Table 5** Peptide biomarker candidates serum quantitation summary by LC-MS/MS

|  |  |  |  |
| --- | --- | --- | --- |
|  | Peptide serum concentration (mean±standard deviation, ng/ml) | | |
|  | ITIH4-2026.9 | FGA-1033.4^d^ | ITIH4-2051.1 |
| **Discovery cohort (n=115)** |  |  |  |
|  |  |  |  |
| PE-positive (n=30) | 422.23±61.58 | 0.50±0.23 | 49.23±11.86 |
| PE-negative subgroup (n=30) | 216.93±78.58 | 0.08±0.21 | 38.34±13.37 |
| PE-negative total (n=85) | 224.29±103.55 | -0.06±0.30 | 32.01±15.29 |
| *p*^a^ | <0.001 | <0.001 | <0.001 |
| *p*^b^ | <0.001 | <0.001 | <0.001 |
| **Validation cohort (n=80)** |  |  |  |
|  |  |  |  |
| PE-positive (n=20) | 384.39±87.75 | 0.37±0.33 | 44.99±18.08 |
| PE-negative (n=60) | 237.09±95.31 | -0.10±0.26 | 30.00±13.49 |
| *p*^c^ | <0.001 | <0.001 | 0.002 |
| a: comparison between PE-positive (n=30) and PE-negative subgroup (n=30) of the discovery cohort; b: comparison between PE-positive (n=30) and PE-negative (n=85) of the discovery cohort; c: comparison between PE-positive (n=20) and PE-negative (n=60) of the validation cohort; d: the serum concentration of FGA-1033.4 was log-tranformed to reach normal distribution. | | | |

**Supplementary Figures**

**Supplementary Figure 1** Chromatograms of the peptide candidates in clinical serum samples with or without synthesized isotope-labeled standard peptides spike. FGA-1033.4 (A), ITIH4-2026.9 (B), ITIH4-2051.1 (C), C3-1876.9 (D).

**Supplementary Figure 2** Calibration curves of the peptide candidates by LC-MS/MS. ITIH4-2026.9 (A), FGA-1033.4 (B), ITIH4-2051.1 (C).

**Supplementary Figure 3** Three-dimensional scattered plots for the peptide candidates in the discovery (A) and validation (B) cohorts. The x-, y- and z-axis represent ITIH4-2026.9, FGA-1033.4, and ITIH4-2051.1 respectively. Red dots: PE-positive patients; blue dots: PE-negative patients.

**References**

1. Jia K, Li W, Wang F, Qu H, Qiao Y, Zhou L, Sun Y, Ma Q, Zhao X. Novel circulating peptide biomarkers for esophageal squamous cell carcinoma revealed by a magnetic bead-based MALDI-TOFMS assay. *Oncotarget*. 2016;7(17):23569-80. doi:10.18632/oncotarget.8123
2. Liu Y, Wei F, Wang F, Li C, Meng G, Duan H, Ma Q, Zhang W. Serum peptidome profiling analysis for the identification of potential biomarkers in cervical intraepithelial neoplasia patients. *Biochem Biophys Res Commun*. 2015;465(3):476-80. doi:10.1016/j.bbrc.2015.08.042
3. Zheng H, Li R, Zhang J, Zhou S, Ma Q, Zhou Y, Chen F, Lin J. Salivary biomarkers indicate obstructive sleep apnea patients with cardiovascular diseases. *Sci Rep*. 2014;4:7046. doi:10.1038/srep07046
4. Campbell J, Rezai T, Prakash A, Krastins B, Dayon L, Ward M, Robinson S, Lopez M. Evaluation of absolute peptide quantitation strategies using selected reaction monitoring. *Proteomics*. 2011;11(6):1148-52. doi:10.1002/pmic.201000511
