# Supplementary figures and images for: Serum peptidomic screening identified circulating peptide biomarkers predictive for preeclampsia
Source: Front Cardiovasc Med. 2022 Oct 11;9:946433. doi: 10.3389/fcvm.2022.946433 (PMC9595599; doi:10.3389/fcvm.2022.946433)

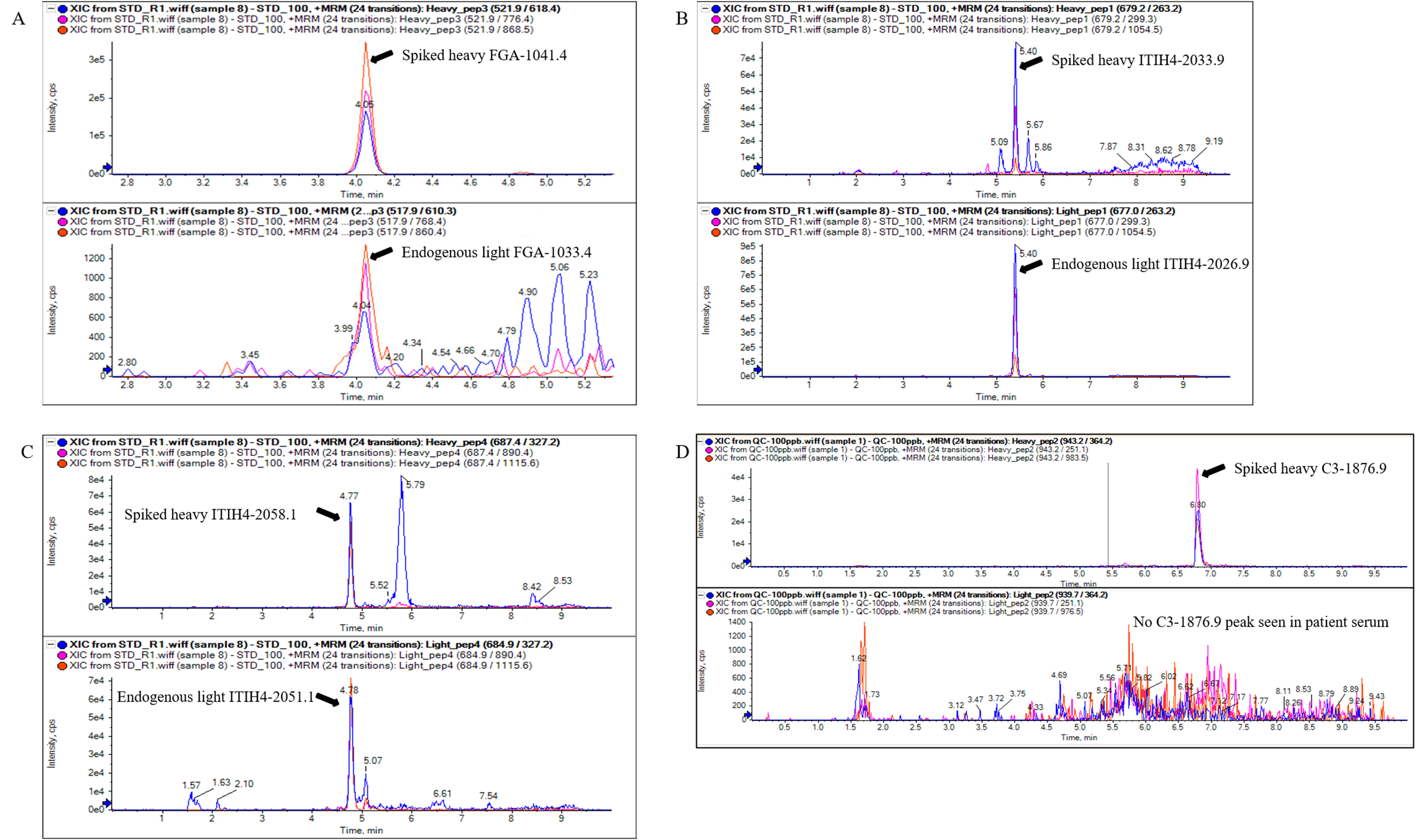

Supplement: Supplementary file 2 [file Image_1.TIF]

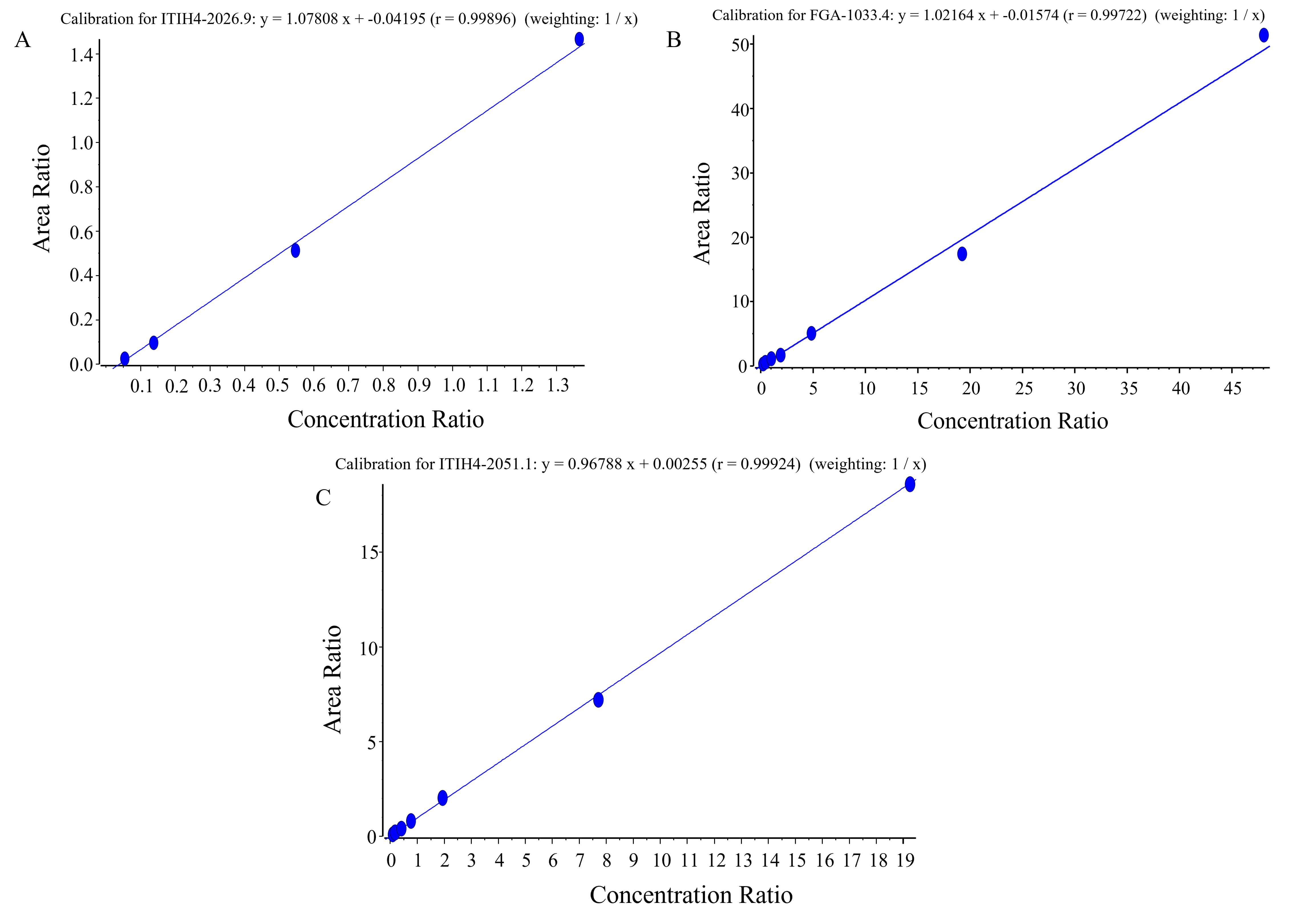

Supplement: Supplementary file 3 [file Image_2.TIF]

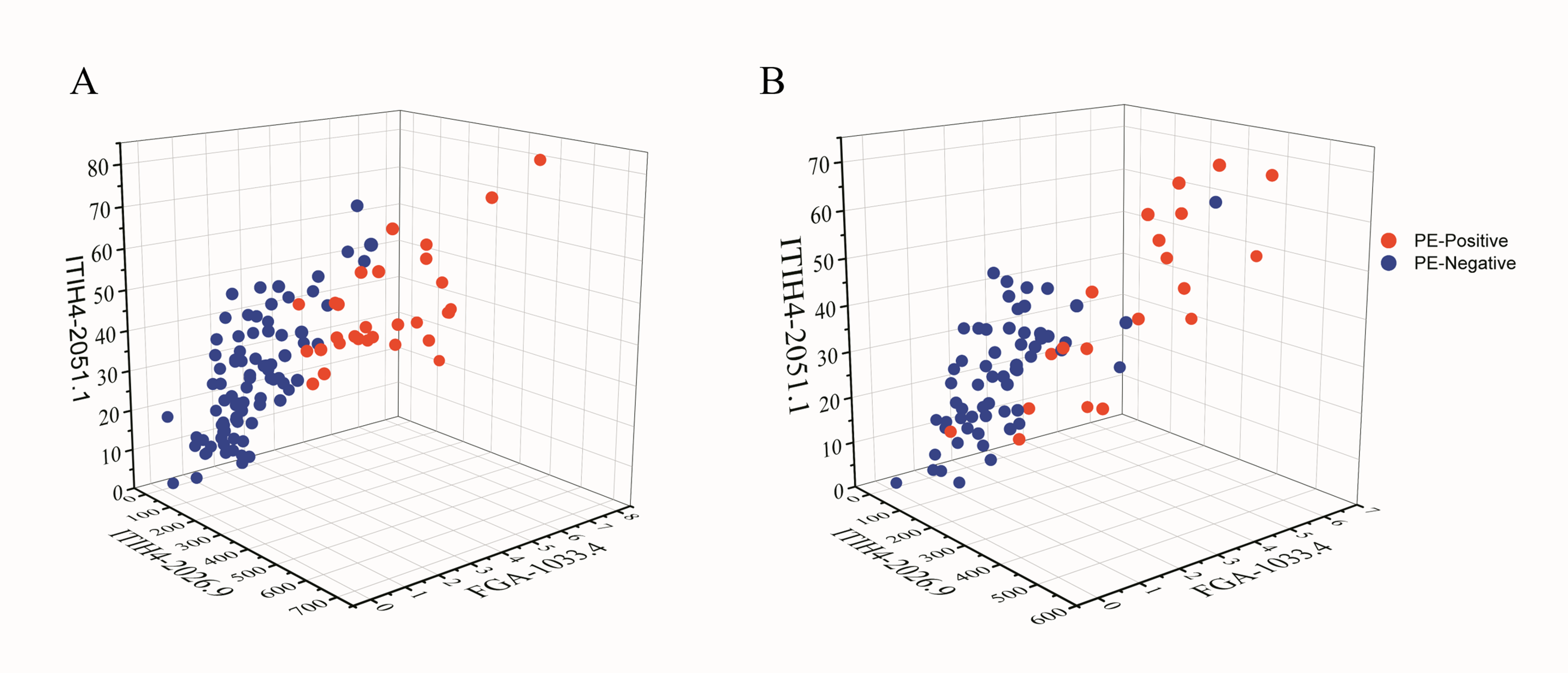

Supplement: Supplementary file 4 [file Image_3.TIF]
